# Reconciling Assumptions in Bottom‐Up and Top‐Down Approaches for Estimating Aerosol Emission Rates From Wildland Fires Using Observations From FIREX‐AQ

**DOI:** 10.1029/2021JD035692

**Published:** 2021-12-10

**Authors:** E. B. Wiggins, B. E. Anderson, M. D. Brown, P. Campuzano‐Jost, G. Chen, J. Crawford, E. C. Crosbie, J. Dibb, J. P. DiGangi, G. S. Diskin, M. Fenn, F. Gallo, E. M. Gargulinski, H. Guo, J. W. Hair, H. S. Halliday, C. Ichoku, J. L. Jimenez, C. E. Jordan, J. M. Katich, J. B. Nowak, A. E. Perring, C. E. Robinson, K. J. Sanchez, M. Schueneman, J. P. Schwarz, T. J. Shingler, M. A. Shook, A. J. Soja, C. E. Stockwell, K. L. Thornhill, K. R. Travis, C. Warneke, E. L. Winstead, L. D. Ziemba, R. H. Moore

**Affiliations:** ^1^ NASA Postdoctoral Program Universities Space Research Association Columbia MD USA; ^2^ NASA Langley Research Center Hampton VA USA; ^3^ Science Systems and Applications, Inc. Hampton VA USA; ^4^ CIRES University of Colorado Boulder Boulder CO USA; ^5^ Earth Systems Research Center University of New Hampshire Durham NH USA; ^6^ National Institute of Aerospace Hampton VA USA; ^7^ Environmental Protection Agency Research Triangle Durham NC USA; ^8^ College of Arts and Sciences Howard University Washington DC USA; ^9^ NOAA Chemical Science Laboratory Boulder CO USA; ^10^ Department of Chemistry Colgate University Hamilton NY USA

## Abstract

Accurate fire emissions inventories are crucial to predict the impacts of wildland fires on air quality and atmospheric composition. Two traditional approaches are widely used to calculate fire emissions: a satellite‐based top‐down approach and a fuels‐based bottom‐up approach. However, these methods often considerably disagree on the amount of particulate mass emitted from fires. Previously available observational datasets tended to be sparse, and lacked the statistics needed to resolve these methodological discrepancies. Here, we leverage the extensive and comprehensive airborne in situ and remote sensing measurements of smoke plumes from the recent Fire Influence on Regional to Global Environments and Air Quality (FIREX‐AQ) campaign to statistically assess the skill of the two traditional approaches. We use detailed campaign observations to calculate and compare emission rates at an exceptionally high‐resolution using three separate approaches: top‐down, bottom‐up, and a novel approach based entirely on integrated airborne in situ measurements. We then compute the daily average of these high‐resolution estimates and compare with estimates from lower resolution, global top‐down and bottom‐up inventories. We uncover strong, linear relationships between all of the high‐resolution emission rate estimates in aggregate, however no single approach is capable of capturing the emission characteristics of every fire. Global inventory emission rate estimates exhibited weaker correlations with the high‐resolution approaches and displayed evidence of systematic bias. The disparity between the low‐resolution global inventories and the high‐resolution approaches is likely caused by high levels of uncertainty in essential variables used in bottom‐up inventories and imperfect assumptions in top‐down inventories.

## Key Points

- In situ measurements of wildland fire smoke plumes provide emission rates for evaluating emissions inventories at unprecedented resolution
- Fire emissions inventories struggle to capture the emissions rate characteristics of individual fires but may perform well in the aggregate
- Bottom‐up inventories suffer from major uncertainty in key variables, while top‐down inventories may have bias from imperfect assumptions

## 1 Introduction

In the Western United States, there is a need for balance between reducing the hazards of wildland fires while maintaining forest health under the influence of a changing climate. Unless we can better understand and predict the deleterious impacts of wildland fire smoke emissions on air quality and human health, it will be nearly impossible for society to respond and adapt to this evolving and complex system. Informed land management policy that utilizes prescribed fires to reduce fuel buildup and reinvigorate ecosystems in order to ultimately minimize smoke exposure for downwind communities necessitates the ability to quantify the composition, magnitude, and transport of smoke (Noss et al., 2006; Schweizer et al., 2018). In the case of accidental or uncontrolled wildfires, the capability to accurately predict smoke transport is necessary to alert sensitive populations and mitigate the overall impact of smoke on human health (Larkin et al., 2009; McKenzie et al., 2006). Atmospheric models rely entirely on databases of fire locations and estimated emissions (so‐called fire emissions inventories) to represent the contribution of fire emissions to downwind atmospheric composition (Wiedinmyer et al., 2006). These inventories exist as both real‐time or operational emissions inventories and retrospective global and regional inventories, depending on their intended purpose. Two distinct approaches are traditionally used to create these emissions inventories: a fuel accounting based “bottom‐up” approach and an energy based “top‐down” approach (Seiler & Crutzen, 1980; Wooster et al., 2005).

Here, we define all energy‐based approaches as top‐down and all fuel accounting based approaches as bottom‐up. However, it should be noted that energy based fire emissions inventories may be categorized as bottom‐up or top‐down according to the nature of the observations from which their emission factors (or coefficients) are derived. Bottom‐up emission factors are derived through laboratory or field experiments at a number of discrete locations representative of different fuel types, and then generalized for use in global or regional applications, whereas top‐down emission factors are based on global or regional measurements of the appropriate emissions species such as aerosols from satellite, and are used to constrain emissions locally, regionally or globally.

The bottom‐up, fuel accounting based approach calculates the mass of carbon emitted by a fire as the product of burned area, fuel mass per unit area, the carbon fraction of fuel, and combustion completeness (Seiler & Crutzen, 1980). This approach, also known as the carbon mass balance method, most commonly operates under the explicit assumption that all burnt biomass carbon is volatilized and emitted to the atmosphere (Surawski et al., 2016; Ward & Radke, 1993). Although a fuels‐based approach is the key feature of bottom‐up algorithms, most also rely on remote sensing observations from satellite sensors such as the Moderate Resolution Imaging Spectroradiometer (MODIS) or Visible Infrared Imaging Radiometer Suite (VIIRS) to determine burned area. Burned area can be calculated using active fire detections, by assuming the entire landscape captured in the resolution of a single satellite pixel burned, or burned area can be taken directly from higher level data products (Kaiser et al., 2012; Van Der Werf et al., 2017; Wiedinmyer et al., 2011). Fuel mass per unit area is often derived from either a biogeochemical model initialized with satellite observations and/or from fuel databases of fuel type and loading (McKenzie et al., 2012; Pettinari & Chuvieco, 2016; Sandberg et al., 2001; Van Der Werf et al., 2017). Fuel carbon content is often assumed based on laboratory measurements from previous studies or estimated using the sum of CO_2_, CO, and CH_4_ emission factors (Akagi et al., 2011; McMeeking et al., 2009; Santín et al., 2015; Susott et al., 1991; Van Der Werf et al., 2017; Yokelson et al., 1997). Combustion completeness is calculated as a function of changes in landscape characteristics, fuel moisture, summer land surface temperature, tree cover, and/or daily fire weather indices (Kaiser et al., 2012; Michalek et al., 2000; Ottmar, 2014; Van Der Werf et al., 2017; Wiedinmyer et al., 2011).

The bottom‐up approach requires an ecosystem‐specific emission factor to convert total carbon mass emissions to emissions of a particular trace gas or aerosol species (Akagi et al., 2011; Andreae & Merlet, 2001). Emission factors are often attained from compilations of previous studies categorized by fuel or vegetation type and show a wide range of natural variability depending on the exact composition of fuel being burned and combustion conditions (Prichard et al., 2020). Certain species, including many volatile organic compounds (VOCs) and aerosols, rapidly evolve in the atmosphere following emission, which necessitates emission factor estimates derived only from measurements of young, fresh smoke (Garofalo et al., 2019). There are numerous particulate mass (PM) emission factors published from in situ ground measurements of prescribed burns and laboratory based studies, however in situ airborne measurements of PM emission factors for Western US wildland fires are particularly scarce (Akagi et al., 2011).

The top‐down or energy based approach follows from Wooster et al. (2005), who showed that the burning of dry vegetation yields the same amount of energy, regardless of fuel type. Top‐down inventories assume fire radiative power (FRP) observations from satellite remote sensing can be used as a direct measurement of the amount of biomass consumed in a fire in an effort to bypass the latency and uncertainty associated with variables required in bottom‐up style inventories (Ichoku & Kaufman, 2005). In the top‐down approach, FRP is multiplied by a predetermined coefficient, known as a smoke emission coefficient (*C*_*e*_), to calculate fire emission rates of PM. Smoke emission coefficients are constants derived for individual ecosystems by combining multiple years of aerosol optical depth (AOD) remote sensing observations with a mass extinction efficiency (MEE), a constant that relates particle extinction to particle mass (Giglio et al., 2006; Ichoku et al., 2008; Kaiser et al., 2012). FRP and AOD observations from the MODIS instrument operating on the polar orbiting Terra and Aqua satellites are commonly used in top‐down approaches. MODIS provides daily global coverage of FRP observations at 1 km resolution and AOD observations at 3 and 10 km resolutions at nadir (Freeborn et al., 2014; Remer et al., 2005; Wei et al., 2020). Ichoku et al. (2008) demonstrated that the relationship between fire radiative energy (FRE or temporally integrated FRP) and total PM emissions could be quantified using AOD during a controlled laboratory‐based experiment where multiple fuel types were burned and measurements were collected over the duration of each fire. The smoke emission coefficient determined from the laboratory‐based experiment agrees with independent estimates derived from satellite measurements of FRP and AOD measured over large‐scale wildfires, which leads to the assumption that this approach can be extrapolated to global scale observations of FRP and AOD (Ichoku et al., 2008).

There are dozens of top‐down and bottom‐up emissions inventories available for use in atmospheric transport models. These inventories encompass wide ranges of spatial and temporal scales and can be used to account for hundreds of individual pollutants emitted by fires (Darmenov & da Silva, 2013; Ichoku & Ellison, 2014; Kaiser et al., 2009; Mota & Wooster, 2018; Van Der Werf et al., 2017; Wiedinmyer et al., 2011). The choice of which inventory to use in modeling applications is crucial, because different fire emissions inventories can profoundly disagree on the magnitude, composition, and temporal variability of fire emissions, especially PM (Carter et al., 2020; Larkin et al., 2014; Liu et al., 2020; Pan et al., 2020). The underlying cause of the disagreement is difficult to isolate, but could be due to uncertainty or error in the various assumptions used in each inventory. Most global emissions inventories are plagued with high levels of uncertainty stemming from the individual datasets used to calculate emissions, which further complicates the ability to isolate the cause of the discrepancies among inventories (French et al., 2004; Urbanski et al., 2011; Wiedinmyer et al., 2011). For example, the detection and quantification of active fire locations, FRP, and AOD using satellite remote sensing suffers from the obscuration of the land surface by clouds or thick smoke, limited spatiotemporal coverage or resolution, and instrument detection limits.

It is fundamentally challenging to correctly quantify biomass burning emissions due to the highly variable composition and structure of the fuels that fires consume, and because fires can rapidly change their behavior in response to dynamic meteorological or environmental conditions (Kennedy et al., 2020; Liu, 2004; Prichard et al., 2019; Schultz et al., 2008). The datasets used in global fire emissions inventories attempt to capture these dynamics, but they often lack the spatial and temporal resolution needed to fully encapsulate all of the individual components that influence emissions. Intensive measurements of smoke from the joint NASA/NOAA Fire Influence on Regional to Global Environments and Air Quality (FIREX‐AQ) campaign that was conducted during the summer of 2019 provide a unique opportunity to evaluate the assumptions and uncertainties in both top‐down and bottom‐up approaches for calculating fire emissions. During FIREX‐AQ, the NASA DC‐8 aircraft was outfitted with a comprehensive instrument payload that sampled smoke plumes from Western US wildland fires and Southeastern US prescribed and agricultural fires. The plume sampling strategy for the western portion of the campaign consisted of an above‐plume, longitudinal run along the entire length of the plume to allow for nadir‐pointing remote sensing of the smoke followed by a set of plume transects perpendicular to the direction of smoke transport where the aircraft sampled the plume in situ during a series of sequentially downwind, cross‐sectional passes (Wiggins et al., 2020).

Measurements collected during FIREX‐AQ provide the opportunity for a rare direct comparison and evaluation of the traditional, lower resolution approaches to calculate fire emissions at an unusually high spatial and temporal resolution. We utilize FIREX‐AQ smoke plume measurements to calculate total carbon and total PM emission rates from Western US wildland fires using three separate high‐resolution approaches. We first calculate fire emission rates from fires sampled during FIREX‐AQ using a novel, independent approach based on direct observations. We further capitalize on FIREX‐AQ data to calculate fire emission rates using a high‐resolution top‐down approach and a high‐resolution bottom‐up approach. The high‐resolution top‐down approach (referred to as HSRL‐GOES) uses airborne HSRL measurements of particle extinction to calculate aerosol optical thickness (AOT) instead of satellite observations of AOD, and the high‐resolution bottom‐up approach (referred to as Fuel2Fire) uses carbon emission estimates from the newly developed Fuel2Fire carbon emissions inventory that has been developed and optimized specifically to estimate emissions from the fires sampled during FIREX‐AQ. We also obtain emission rates from a traditional bottom‐up fire emissions inventory, Global Fire Emissions Database (GFED4.1s), and a traditional top‐down fire emissions inventory, Fire Energetics, and Emissions Research (FEERv1.0). GFED and FEER have much lower temporal and spatial resolutions (3‐hr/daily at 0.25° and daily at 0.1°, respectively) compared to the three high‐resolution FIREX‐AQ based approaches.

**Figure 1:**
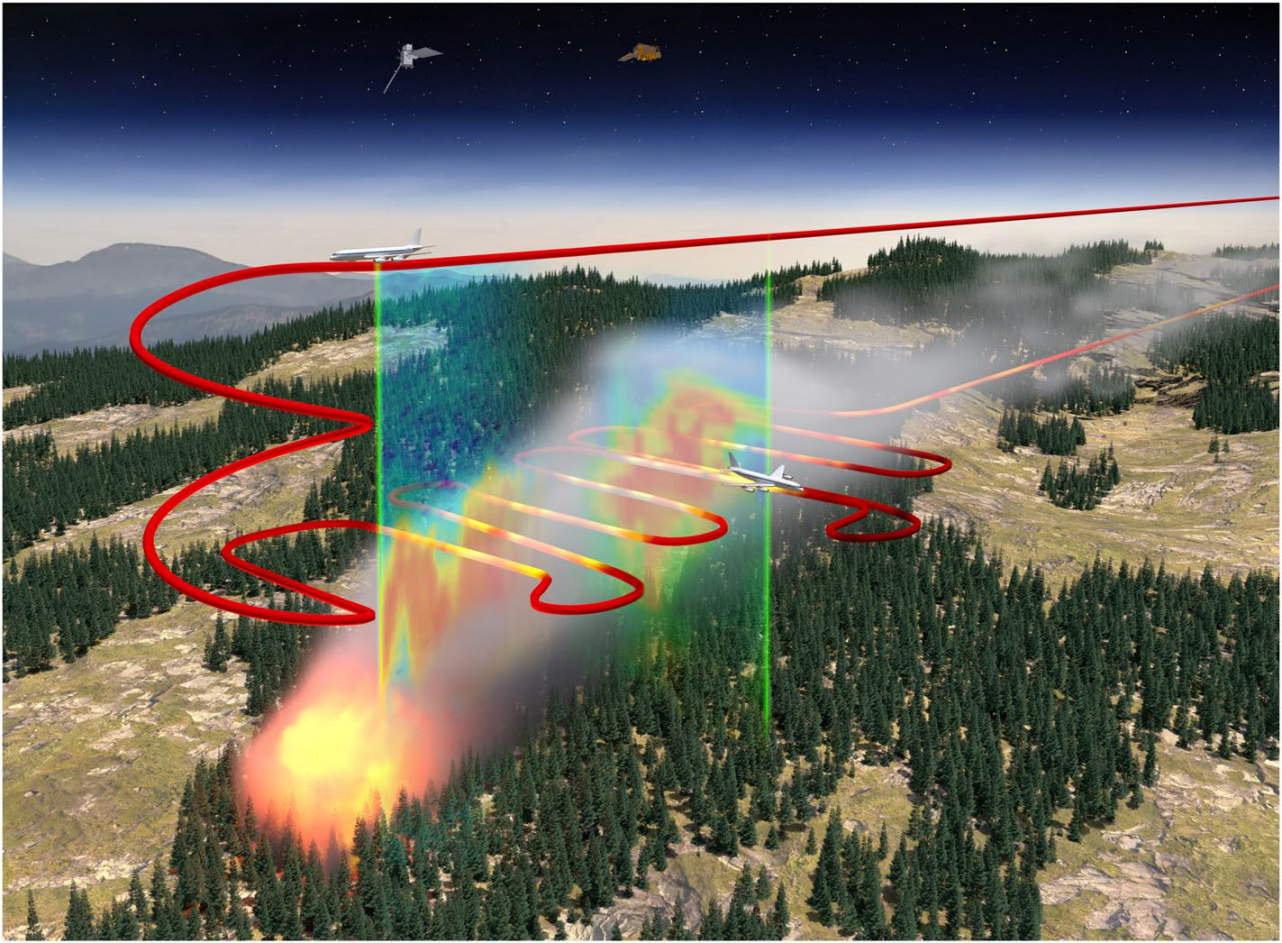
Conceptual image of a typical wildland fire and smoke plume observed during Fire Influence on Regional to Global Environments and Air Quality (FIREX‐AQ) as well as the observational platforms and analysis approaches. The DC‐8 flight track is given in red and colored by in situ particle concentrations for the cross‐sectional legs. As described in the text, the DC‐8 initially completes a longitudinal run where the nadir High‐Spectral Resolution Lidar (HSRL) measurement provides the full smoke curtain below the aircraft, which is then followed by a series of successively downwind flight legs where the nadir‐ and zenith‐pointing HSRL curtains are used to contextualize the cross‐sectional, in situ measurements. Image credit: NASA/Tim Marvel.

We evaluate the performance of GFED and FEER, along with the high‐resolution top‐down and bottom‐up approaches, against the in situ measurement based approach to investigate potential bias and assess the validity of the assumptions unique to each approach (Figure 1). We also investigate and quantify uncertainty for all of the approaches used to calculate emission rates in this study. The goal of this paper is to understand how the estimates of total carbon and total PM emission rates from traditional, lower resolution methods compare to the high‐resolution estimates available for the fires sampled during the FIREX‐AQ campaign. The results of this analysis should be of keen interest for the global wildfire emissions inventory community as well as atmospheric scientists seeking to use airborne observations to constrain wildland fire aerosol emissions.

## 2 Methods

### 2.1 Emission Rate Estimates From Global Inventories

#### 2.1.1 GFED4.1s (Low‐Resolution Bottom‐Up)

GFED is a global fire emissions inventory that internally calculates carbon emission rates using a traditional bottom‐up (fuels‐based) approach as follows

(1) $$E_{C}=\text{BA}\times \text{FL}\times \text{CC}\times F_{C}$$

where *E*_*C*_ is the carbon mass emission rate per day, BA is the burned area, FL is the fuel mass loading per area, CC is the combustion completeness (expressed as a percent), and *F*_*C*_ is the mass fraction of carbon in the fuel (Van Der Werf et al., 2017). GFED obtains burned area estimates from MODIS (MCD64A1), fuel loading and combustion completeness are derived from the Carnegie‐Ames‐Stanford Approach (CASA) biogeochemical model, and carbon mass fraction is defined per ecosystem from compilations of previous studies (Akagi et al., 2011; Andreae & Merlet, 2001; Van Der Werf et al., 2017).

To represent a traditional bottom‐up approach, we use daily average carbon emission rates per area from GFED4.1s (https://www.globalfiredata.org/) to calculate daily average PM emission rates (*E*_PM_) for the western fires sampled during FIREX‐AQ as follows

(2) $$E_{\text{PM}}={\text{EF}}_{\text{PM}}\times \sum \frac{{\hat{E}}_{C}\times A_{P,\text{GFED}}\;}{F_{c}}$$

where EF_PM_ is the total particulate matter mass emission factor suggested by GFED for temperate forests (17.6 gPM kg‐biomass consumed^−1^), ${\hat{E}}_{C}$ is the area‐normalized daily carbon emissions in each GFED pixel, *A*_P,GFED_ is the GFED pixel area (0.25° × 0.25°), and the summation is carried out over all GFED pixels within 0.25° of the centroid of the final fire perimeter for each fire. The final fire perimeters were derived from the United States Geological Survey Geospatial Multi‐Agency Coordination Group (GeoMAC) mapping application hosted by the National Interagency Fire Center (https://data-nifc.opendata.arcgis.com/datasets/historic-perimeters-2019) (Walters et al., 2011). In Equation 2, we use the *F*_*C*_ suggested by GFED for temperate forests (0.489 kgC kg‐biomass consumed^−1^). The use of ecosystem level constant values for EF_PM_ and *F*_*C*_ is intended to provide good results in aggregate on the regional‐to‐global scales required by models, although individual fires will deviate from these specifications. GFED data are provided on a daily and a 3‐hr basis in UTC time, and here, we use the daily product. We convert from UTC time to local time by assuming daily emissions (local time) are equal to 75% of the emissions from the day a given fire was sampled by the DC‐8 aircraft (local time) plus 25% of the emissions from the day after (local time).

We estimate relative uncertainty in *E*_*C*_ and *E*_PM_ estimates derived from GFED by propagating uncertainty through Equations 1 and 2. For Equation 1, we assume the following relative uncertainties: BA = 44%, FL = 111%, CC = 11%, and *F*_*C*_ = 10%. For Equation 2, we assume EF_PM_ has a relative uncertainty of 36% and *E*_*C*_ has a relative uncertainty calculated by propagating uncertainty through Equation 1. We obtain the relative uncertainty in the BA product used by GFED from an analysis of MODIS burned area by Giglio et al. (2018). FL and CC relative uncertainty are derived by taking the standard deviation divided by the average for all field measurements of Western US fuels as compiled by van Leeuwen et al. (2014) and updated by Van Der Werf et al. (2017). *F*_*C*_ relative uncertainty is defined as the standard deviation divided by the average in *F*_*C*_ values given by Akagi et al. (2011). The relative uncertainty in EF_PM_ is calculated as the standard deviation divided by the average of EF_PM_ derived from all previous studies of temperate forest EF_PM_ measurements used in GFED (Akagi et al., 2011; Andreae & Merlet, 2001; Van Der Werf et al., 2017). The calculated relative uncertainty in GFED *E*_PM_ is 126% and *E*_*C*_ is 120% (Table S1).

#### 2.1.2 FEERv1.0 (Low‐Resolution Top‐Down)

FEER is a global fire emissions inventory that calculates daily average *E*_PM_ using a traditional top‐down (energy‐based) approach as

(3) $$E_{\text{PM}}=C_{e}\times \text{FRP}$$

where *C*_*e*_ is an ecosystem‐dependent predetermined smoke emission coefficient, and FRP observations are from MODIS. FEER derives *C*_*e*_ using multiple years of coupled MODIS AOD (550 nm) and FRP observations and an assumed constant MEE at 550 nm of 4.6 m^2^ g^−1^ derived from previous studies (Reid, Eck, et al., 2005). Smoke emission coefficients have been predetermined by FEER and are provided globally at a 1° × 1° resolution (https://feer.gsfc.nasa.gov/projects/emissions/) (Ichoku & Ellison, 2014).

Daily average *E*_PM_ estimates are provided at a 0.1° × 0.1° resolution through a coupling of FEER smoke emission coefficients and MODIS FRP observations (FEERv1.0‐G1.2). For the traditional top‐down approach, we calculate daily average *E*_PM_ estimates for each of the western fires sampled during FIREX‐AQ as

(4) $$E_{\text{PM}}=\sum {\hat{E}}_{{\text{PM}}_{\text{FEER}}}\times A_{P,\text{FEER}}$$

where ${\hat{E}}_{{\text{PM}}_{FEER}}$ is the area‐normalized daily average *E*_PM_ from each FEER pixel, and $A_{P,FEER}$ is the FEER grid cell area (0.1° × 0.1°). The summation is over all FEER grid cells per fire. FEER grid cells are included if they are within 0.1° of the centroid of the final GeoMAC fire perimeter for each fire. We convert from UTC time to local time following the same approach described in Section 2.1.1. We also calculate the average FEER *C*_*e*_ for the fires sampled during the western portion of the FIREX‐AQ campaign as the average of all *C*_*e*_ estimates in every 1° grid cell that encompassed at least a fraction of the final GeoMAC perimeter of a fire.

We estimate relative uncertainty in FEER *E*_PM_ estimates by propagating uncertainty through Equation 3. We calculate the relative uncertainty of *C*_*e*_ as the standard deviation divided by the mean of all extracted FEER *C*_*e*_ values used in this analysis, and we obtain the relative uncertainty of MODIS FRP from Freeborn et al. (2014). FEER *C*_*e*_ for Western US wildland fires has a relative uncertainty of 73% and MODIS FRP has a relative uncertainty of 27%, yielding a relative uncertainty in FEER *E*_PM_ of 78% (Table S1).

### 2.2 Emission Rate Estimates From FIREX‐AQ

#### 2.2.1 In Situ Measurement Approach (High Resolution)

We capitalize on the intensive, high spatial and temporal resolution smoke plume measurements from the DC‐8 aircraft during FIREX‐AQ to calculate *E*_C_ and *E*_PM_ via a novel in situ measurement‐driven approach. Here, we integrate in situ trace gas and aerosol measurements with information on plume thickness derived from airborne High‐Spectral Resolution Lidar (HSRL) measurements to calculate emission rates. Although this new approach is subject to its own uncertainties and sources of error, we assume emission rate estimates derived from this approach are as close to accurate as we can realistically achieve, because they are based on in situ measurements, and their calculation does not require as many assumptions as the traditional approaches. We assume fire emission rates over time are equal to the flux of smoke as it passes through a vertical slice of the smoke plume, represented as an HSRL curtain measured during in situ transects (Figure 1). We calculate *E*_*C*_ and *E*_PM_ for each wildland fire sampled during FIREX‐AQ on a sub‐plume (per transect) basis as

(5) $$E_{X}=\bar{\text{WS}}\times \bar{\text{GS}}\times \sum_{t_{\text{start}}}^{t_{\text{end}}}\Delta X_{t}\times H_{t}\Delta t$$

where *E*_*x*_ is the emission rate of species *X* (either carbon or PM), $\bar{\text{WS}}$ is the transect average wind speed, $\bar{\text{GS}}$ is the transect average ground speed, $\Delta X_{t}$ is the excess concentration of species *X* averaged over 10 s intervals to match the horizontal resolution of the HSRL data collected at aircraft measurement time *t*, and *H*_*t*_ (m) is the plume thickness measured by the nadir and zenith pointing HSRL profiles at aircraft measurement time *t* (Hair et al., 2008). This approach assumes that the vertical distribution of each species is uniform, and the lidar is used to find the vertical extent of the plume. Horizontal plume boundaries are defined as having a minimum enhancement in CO of 200 ppbv above background concentration. The ground speed averaged across all fires and all transects is 152 ± 11 m/s. Excess concentrations are calculated by subtracting a background concentration defined as the average concentration 5–10 s prior to the start of the transect and 5–10 s after the end of the transect. In a few cases, the PM background is elevated during the time interval used to define a background such that the excess mixing ratio is computed as a negative value, and these cases are excluded from the analysis. The time interval from *t*_start_ to *t*_end_ is equal to the length of time to complete each transect, and Δt is ∼10 s, which is the horizontal resolution of the HSRL data. *H_t_* is calculated as the sum of HSRL profile bin heights ($\Delta z_{t}$) where the particle backscatter coefficient at 532 nm ($\beta_{t}$) is greater than 1 km^−1^ sr^−1^, which was larger than the average background scattering and defined the smoke plume edges for the cases sampled.

(6) $$H_{t}=\sum_{z=0}^{z=\infty }\Delta z_{t}\left[\beta_{t}>1{\;\text{km}}^{-1}\;{\text{sr}}^{-1}\right]\;$$

In exceptionally dense smoke plumes, the HSRL laser light was fully attenuated before it could completely pass through the smoke plume edge, and for these cases we assume the smoke plume extended to the surface and neglect the missing portion of the plume above the aircraft. This approach is reasonable as the aircraft tended to sample the smoke plumes near the top of the atmospheric boundary layer, which places a weak upper constraint on the top of the plume just as the surface places a lower constraint on boundary layer mixing processes. An alternative approach to calculate plume thickness using HSRL observations leverages the ratio of the backscatter coefficient in a single HSRL bin to the sum of all backscatter coefficients in a vertical column. We estimate the sensitivity of plume thickness to these two approaches and discover strong agreement (slope = 0.72, *r* = 0.83), although the alternative approach estimates slightly lower plume thickness on average (Figure S1). We ultimately choose to calculate plume thickness as outlined in Equation 6 in an effort to avoid additional uncertainty from relying more heavily on the backscatter coefficient, which may be confounded by changes in aerosol size and/or optical properties rather than mass loading.

Total PM is calculated as the sum of organic aerosol (OA), sulfate, nitrate, ammonium, and black carbon aerosol (BC) reported at standard temperature and pressure conditions and converted to ambient volumetric units. All components of the submicron non‐refractory total PM concentrations are measured using an Aerodyne Time of Flight Aerosol Mass Spectrometer (ToF‐AMS) (Canagaratna et al., 2007; DeCarlo et al., 2006; Guo et al., 2021). Refractory BC mass concentrations are provided by a Single Particle Soot Photometer (SP2, Droplet Measurement Technologies) (Gao et al., 2007). The 50% geometric transmission diameter for the ToF‐AMS is ∼600 nm, which sufficiently captures the size range for the majority of biomass burning derived particles, with the exception of supermicron ash particles (Adachi et al., 2021; Moore et al., 2021). Total carbon is calculated as the sum of CO_2_, CO, CH_4_, organic carbonaceous aerosol (OC), and BC aerosol. OC is estimated using the OA to OC ratio provided by the ToF‐AMS. The CO_2_ mixing ratio measurements are obtained using a non‐dispersive infrared (IR) spectrometer (LICOR, Inc. Model 7000) adapted for aircraft measurements in a method similar to Vay et al. (2003), while CO and CH_4_ mixing ratios are obtained from mid‐IR laser absorption spectrometry (Sachse et al., 1991). All three trace gas species were calibrated in‐flight with standards from the National Oceanic and Atmospheric Administration Earth Science Research Laboratories (NOAA ESRL) traceable to World Meteorological Organization (WMO) scales. The trace gas measurements were converted from mole fractions to ambient volumetric units by multiplying the mixing ratio by the ratio of the molecular weight to the molecular volume at ambient temperature and pressure conditions.

We estimate relative uncertainty in *E*_*C*_ and *E*_PM_ using Equations 5 and 6. We calculate the following relative uncertainties: WS = 20%, GS = 3%, *H*_*t*_ = 28%, Δ*C* = 56%, and ΔPM = 67%. The relative uncertainty for each variable is assumed to be equal to the mean divided by the standard deviation of observations collected during all smoke plume transects. The computed relative uncertainty in *E*_*C*_ is 66% and the relative uncertainty in *E*_PM_ is 75% (Table S1). Variability in background mixing ratios was negligible relative to the plume enhancements and is neglected.

#### 2.2.2 Fuel2Fire (High‐Resolution Bottom‐Up)

*E*_*C*_ estimates for all FIREX‐AQ wildland fires derived using a bottom‐up style approach are publicly available on the FIREX‐AQ data archive under the analysis tab (https://www-air.larc.nasa.gov/cgi-bin/ArcView/firexaq?ANALYSIS=1#SOJA.AMBER/) Additional detail concerning the Fuel2Fire methodology for calculating carbon emissions is available in the header of the individual data files. This data set, the Fuel2Fire carbon emissions inventory, is optimized and designed to estimate carbon emissions specifically for the fires sampled during FIREX‐AQ. We use Fuel2Fire *E*_*C*_ estimates for the high‐resolution bottom‐up approach to estimate *E*_*C*_ and *E*_PM_ on a per transect basis for each of the fires included in this analysis. As a bottom‐up inventory, Fuel2Fire calculates *E*_*C*_ in the same way as GFED, following Equation 1. The Fuel2Fire emissions inventory derives burned area using a combination of active fire detections from MODIS, VIIRS, and/or Geostationary Operational Environment Satellite Program (GOES‐16 and 17 ABI L2 +). Active fire pixels from one or more of these active fire detection products are selected to best match ground‐verified interagency situational reports from fire management teams, as well as GeoMAC fire perimeters. Fuel2Fire determines fuel loading using high‐resolution (30 m) fuels data from the Fuels Characteristics and Classification System (FCCS) (https://landfire.gov/fccs.php) (Ottmar et al., 2007) and models combustion completeness as a function of daily fire weather danger ratings. Fire weather danger ratings are derived using the National Fire Danger Rating System (Bradshaw et al., 1984) and obtained by extracting observed fire danger classes using daily maps of fire danger provided by the United States Forest Service Wildland Fire Assessment System (http://www.wfas.net/). Total daily carbon emissions are temporally distributed using a diurnal cycle of fire activity derived from geostationary satellite observations of FRP from GOES‐16 and 17. Fuel2Fire assumes *F*_*C*_ is 0.5 kg kg^−1^, but we note *F*_*C*_ can vary from 0.45 to 0.55 (Akagi et al., 2011; Burling et al., 2010; McMeeking et al., 2009; Santín et al., 2015; Susott et al., 1996; Yokelson et al., 1997). The archived carbon emissions data has a native temporal resolution that matches GOES‐16 and 17 data (5 min) and is linearly interpolated to 1 Hz data for consistency with the aircraft data. *E*_*C*_ estimates from Fuel2Fire extend over the course of an entire 24‐hr day (local time) that a given fire was sampled during FIREX‐AQ.

We convert *E*_*C*_ estimates from the Fuel2Fire inventory to *E*_PM_ as follows

(7) $$E_{PM}=\frac{E_{C}\times \;{\text{EF}}_{PM}}{F_{C}}$$

Here, we obtain *E*_*C*_ from the Fuel2Fire inventory, while EF_PM_ is calculated using aircraft observations of trace gas and aerosol concentrations in smoke plumes from Western US wildland fires measured during FIREX‐AQ. We choose to calculate EF_PM_ from in situ observations as opposed to assuming EF_PM_ from a compilation of previous studies in order to investigate the potential influence of the choice in EF_PM_ on differences in emission rate estimates. We calculate EF_PM_ for each in situ smoke plume transect using airborne measurements following the carbon mass balance approach (Ward & Radke, 1993; Yokelson et al., 1996, 1999). The time of emission is not the same as when the DC‐8 sampled the plume, so we correct for this time offset by adding smoke age to the time of emission when determining the Fuel2Fire total carbon emission rates on a subplume, per transect basis. The smoke age is calculated for each point on the DC‐8 transect assuming horizontal straight line advection of the smoke plume at the DC‐8 measured wind speed (Wiggins et al., 2020). Although the smoke age and, thus, probability of plume processing increases as a function of downwind distance from the fire, we assume PM is conserved over the relatively short period of time (0.5–7 hr) that the smoke has been exposed to atmospheric aging processes when it was sampled by the DC‐8 and attribute any changes in mass concentration to variability in fire activity (Garofalo et al., 2019; Hodshire et al., 2019).

We estimate relative uncertainty in *E*_*C*_ and *E*_PM_ derived from Fuel2Fire by propagating uncertainty through Equation 7. The relative uncertainty in *E*_*C*_ is assumed to be 55%, calculated by taking the average divided by the standard deviation of all computed *E*_*C*_ estimates for every fire and every transect included in this analysis. The relative uncertainty in EF_PM_ is 39%, computed as the mean of all calculated EF_PM_ for all fires and all transects divided by the standard deviation. The relative uncertainty in *E*_PM_ is thus 67% (Table S1).

#### 2.2.3 HSRL‐GOES (High‐Resolution Top‐Down)

We use FIREX‐AQ aircraft‐based HSRL measurements of aerosol extinction and geostationary satellite observations of FRP from GOES to calculate *E*_PM_ using a high‐resolution top‐down approach, referred to as HSRL‐GOES. We use the same equation that is used in FEER (Equation 3) to calculate *E*_PM_ for the western fires sampled during FIREX‐AQ on a per transect basis for the high‐resolution top‐down approach. Instead of using MODIS FRP, we obtain FRP from the GOES‐16 and GOES‐17 ABI L2 + Fire/hot spot Detection and Characterization product from the Wildfire Automated Biomass Burning Algorithm processing system (Schmidt, 2019). GOES has an exceptionally high temporal resolution (∼5–15 min) with FRP observations that cover the entire continental US at a spatial resolution of 2 km at nadir (Schmidt, 2019). We time align GOES FRP observations to match the in situ plume sampling time by adding the smoke age to the FRP observation time, and we include all FRP observations within 4 km of a given fire's final GeoMAC perimeter centroid. FRP per transect is calculated as the sum of all instantaneous FRP observations for a given fire averaged over the in situ plume sampling time for a given transect. The smoke emission coefficient (*C*_*e*_) is also calculated for each fire on a per transect basis as follows:

(8) $$C_{e}=\frac{\bar{\text{WS}}\times \bar{\text{GS}}}{\bar{\text{MEE}}\times \bar{{\text{FRP}}_{f}}}\;\times \sum_{t_{\text{start}}}^{t_{\text{end}}}\Delta {\text{AOT}}_{t}\;\Delta t$$

where $\bar{{\text{FRP}}_{f}}$ is the time‐aligned, transect‐average GOES FRP, $\bar{\text{MEE}}$ is the transect average MEE calculated from in situ measurements as described below, and ΔAOT is aerosol optical thickness derived from vertically integrating the background‐subtracted 532 nm HSRL particle extinction coefficient (Δα) as described by

(9) $$\Delta {\text{AOT}}_{t}=\;\int \Delta \alpha_{t}\Delta z$$

HSRL is not able to collect measurements immediately above and below the aircraft. We linearly interpolated through the 60‐m aircraft gap in the HSRL curtains to account for the missing data. Background extinction is defined as the average HSRL extinction profile 10 s before and after the smoke plume transect. In cases, where the laser light fully attenuated before it reached the bottom of the plume, we assume the plume extended to the surface and extrapolate extinction to the ground using the closest measurement to the surface. In limited cases, where the beam is completely attenuated in the zenith direction, we integrate over the measured range but do not add any correction, because this is expected to be a relatively small contribution as the aircraft was typically flying near the top of the atmospheric boundary layer near plume top. We use the high‐resolution in situ measurements from the DC‐8 to calculate MEE; however, we note that most top‐down inventories (such as FEER) assume a constant MEE of 4.6 m^2^ g^−1^ derived from previous studies (Reid, Eck, et al., 2005). We calculate transect average MEE as the slope of a reduced major axis regression with a forced zero intercept between total PM and the dry aerosol extinction coefficient at 532 nm for each transect. The extinction coefficient is calculated as the sum of dry scattering and absorption coefficients measured by a TSI‐3563 Nephelometer at 550 nm and a three‐wavelength Particle Soot Absorption Photometer at 532 nm (PSAP, Radiance Research), respectively. Scattering coefficients are converted to 532 nm to match the absorption coefficients using the angstrom exponent as calculated by the blue and green channels from the nephelometer. Scattering coefficients are corrected for truncation errors following Anderson and Ogren (1998), and PSAP absorption data are corrected following Virkkula (2010). The aerosol extinction humidification factor, *f* (RH) is assumed to be unity, which is consistent with the FIREX‐AQ in‐plume measurements.

We estimate the uncertainty in HSRL‐GOES *E*_PM_ by propagating uncertainty through Equation 3, where the relative uncertainty in *C*_*e*_ derived following Equation 8 is calculated as the mean *C*_*e*_ from all fires and all transects divided by the standard deviation (67%), and the relative uncertainty in FRP is assumed to be 40% (Li et al., 2020). The relative uncertainty in HSRL‐GOES *E*_PM_ is thus 77% (Table S1).

### 2.3 Comparison of Approaches

We summarize the approaches and relevant equations used in this study to calculate *E*_*C*_ and *E*_PM_ in Table 1. We evaluate emission rate estimates between the high‐resolution bottom‐up (Fuel2Fire) and top‐down (HSRL‐GOES) based approaches against the in situ approach on a per transect basis for individual wildland fires sampled during FIREX‐AQ. The relationship between the different approaches is quantified using the slope of a reduced major axis regression with a forced zero intercept, a Pearson's correlation coefficient, and root mean square error (RMSE). These calculations are performed as a campaign level summary that includes all transects and all fires and for each fire individually.

**Table 1:** Summary of Approaches Used to Calculate Fire Carbon and Particulate Mass (PM) Emission Rates

Inventory or approach	Style	Spatial range	Temporal resolution	Eqns.	Input variables	Output variables
GFED4.1s	Bottom‐up	Global	Daily	1	BA, FL, CC, *F*_*C*_	*E*_*C*_
				2	${\hat{E}}_{C_{GFED}}$, EF_PM_, *F*_*c*_, $\Delta X_{\text{GFED}}$	*E*_PM_
FEERv1.0	Top‐down	Global	Daily	3	*C*_*e*_ (MODIS), FRP (MODIS)	*E*_PM_
				4	${\hat{E}}_{PM_{FEER}}$, $\Delta X_{FEER}$	*E*_PM_
In situ	In situ	Western US (FIREX‐AQ)	Subplume timescale (per aircraft transect)	5	CO_2_, CO, CH_4_, OC, BC, PM, H, WS, GS	*E*_*C*_, *E*_PM_
				6	Δz, β	*H*
Fuel2Fire	Bottom‐up	Western US (FIREX‐AQ)	Subplume timescale (per aircraft transect)	1	BA, FL, CC, *F*_*C*_	*E*_*C*_
				7	E_C_, EF_PM_, *F*_*C*_	*E*_PM_
HSRL‐GOES	Top‐down	Western US (FIREX‐AQ)	Subplume timescale (per aircraft transect)	3	C_e_ (Aircraft‐GOES), FRP (GOES)	*E*_PM_
				8	WS, GS, MEE, FRP (GOES), AOT	*C*_*e*_
				9	$\alpha_{t}$, Δz	AOT

*Note*. GFED4.1s also provides data at a 3 hr temporal resolution, but we use only the daily product.

We compare daily (24 hr local time) average *E*_PM_ estimates from the three high‐resolution approaches (In Situ, Fuel2Fire, and HSRL‐GOES) to daily average estimates derived from lower resolution global fire emissions inventories (GFED and FEER). These comparisons are performed on daily average emission rate estimates, as opposed to estimates on a per transect basis, because of the lower temporal resolutions of GFED (3 hr/daily) and FEER (daily).

### 2.4 Smoke Emission Coefficients

The high spatial and temporal resolution of the in situ, bottom‐up (Fuel2Fire), and top‐down (HSRL‐GOES) based approaches provide the opportunity to evaluate smoke emission coefficients that are usually derived using many years of data. Smoke emission coefficients for PM are calculated as the slope of a reduced major axis regression with a forced zero intercept between GOES FRP time aligned to the transect sampling time versus *E*_PM_ for each of the three high‐resolution approaches. These computations are also executed as a campaign level summary and for each fire individually. We compare our *C*_*e*_ from the high‐resolution approaches to the average FEER *C*_*e*_ for the western fires sampled during FIREX‐AQ.

## 3 Results and Discussion

### 3.1 Total Carbon Emission Rates

**Figure 2:**
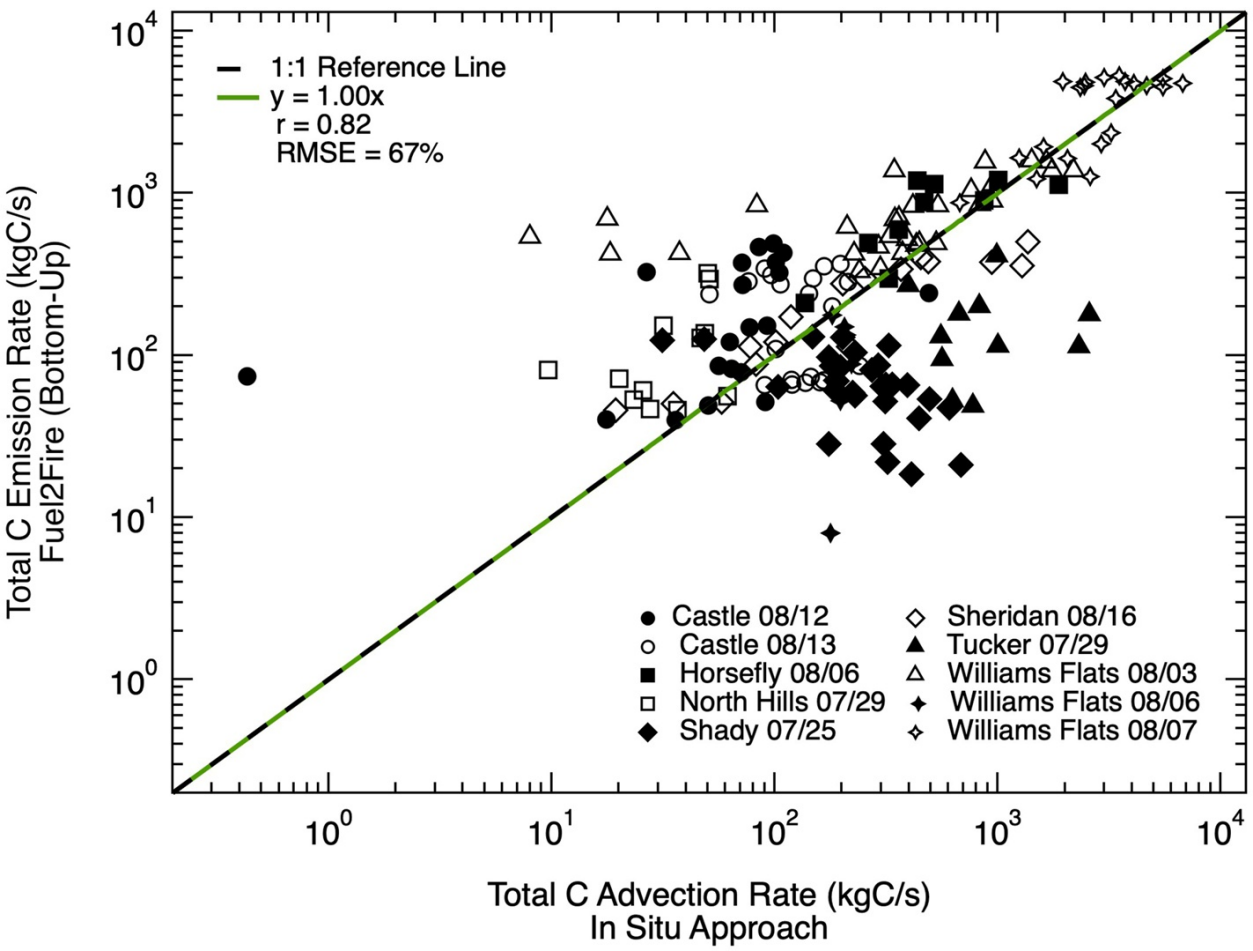
Relationship between total carbon emission rates (*E*_*C*_) from the high‐resolution bottom‐up approach, Fuel2Fire, and the in situ approach. Different markers correspond to specific sampling days for each fire and repeated markers correspond to different transects of the same fire for the given sampling day. The green line shows the fit between *E*_*C*_ using a reduced major axis regression with a forced zero intercept. The dashed black line shows a perfect 1:1 relationship for reference. The slope for the linear fit, Pearson's correlation coefficient (r), and root mean square error (RMSE) are given in the legend.

The derivation of the variables used to calculate *E*_*C*_ using bottom‐up approaches are based on assumptions that can lead to both under and overestimation, depending on the data products leveraged by a given fire emissions inventory. We uncover a significant relationship between *E*_*C*_ per transect derived from the high‐resolution bottom‐up approach (Fuel2Fire) and the in situ approach as shown in Figure 2 (slope = 1.00, *r* = 0.82). However, there is also a nontrivial level of scatter in this relationship (RMSE = 67%), and individual fires considered separately have different correlations and regression slopes.

**Figure 3:**
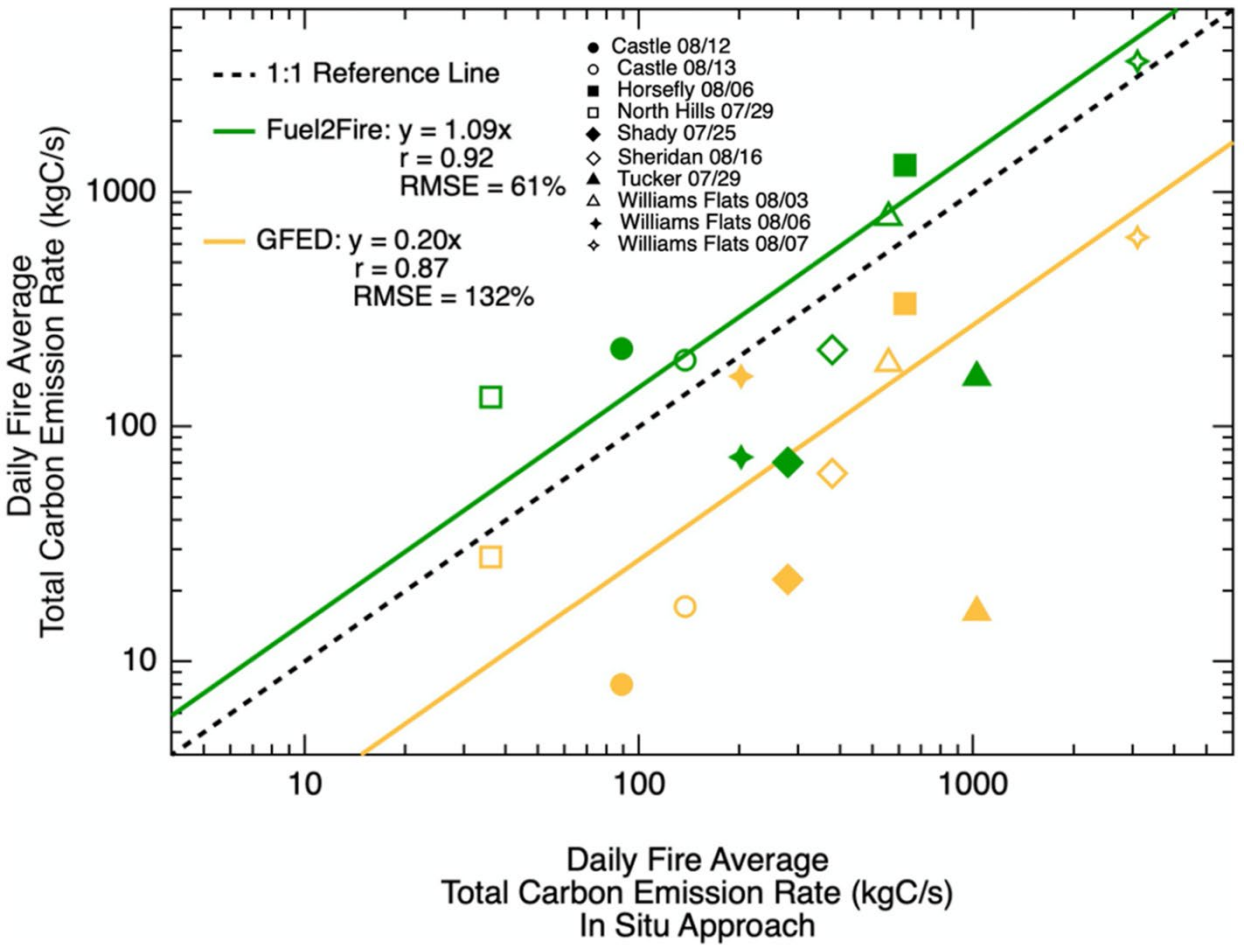
Relationship between daily fire average total carbon emission rates (*E*_*C*_) from Fuel2Fire and GFED versus the in situ measurement based approach. Different markers correspond to specific fires on specific sampling days. The green line shows the fit between Fuel2Fire *E*_*C*_ estimates versus the in situ approach using a reduced major axis regression with a forced zero intercept. The yellow line shows the fit between GFED *E*_*C*_ estimates versus the in situ approach. The dashed black line shows a perfect 1:1 relationship for reference. The slope for the linear fit, Pearson's correlation coefficient (*r*), and root mean square error (RMSE) are given in the legend.

From Figure 3, we similarly find strong, linear correlations between daily fire average *E*_*C*_ from the in situ measurement based estimates and Fuel2Fire (slope = 1.09, *r* = 0.92, RMSE = 61%) and GFED (slope = 0.20, *r* = 0.87, RMSE = 132%). The daily average *E*_*C*_ estimated using Fuel2Fire are marginally higher than estimates derived from the in situ approach, while the GFED estimates are 80% lower. The strong correlation, but significant offset between GFED and the in situ measurement based approach implies that there may be a systematic bias in one or more of the variables used to calculate the mass of biomass consumed in some traditional bottom‐up inventories. In this section, we examine the assumptions and uncertainty in individual variables used to calculate *E*_*C*_ using a bottom‐up approach in an effort to understand the differences in *E*_*C*_ estimates derived from Fuel2Fire and GFED relative to the in situ approach.

#### 3.1.1 Carbon Mass Balance

The key assumption in many bottom‐up approaches is that all burnt carbon is volatilized and released into the atmosphere. This carbon mass balance assumption has recently been scrutinized because not all fuel that has been thermally altered by a fire is emitted to the atmosphere (Santín et al., 2015; Surawski et al., 2016). Some of the burnt fuel remains on the ground as charred biomass. If the carbon mass balance assumption does not hold, then this could potentially cause an overestimation of carbon emissions derived from bottom‐up approaches by up to 50% in temperate forests, depending on levels of combustion completeness (Santín et al., 2015). Our results do not show significant evidence of bias in *E*_*C*_ estimates from Fuel2Fire, but do show a distinct low bias in estimates from GFED. This suggests there are underlying confounding factors to disentangle before it is possible to determine, if the assumptions inherent in the carbon mass balance approach are responsible for a significant bias in bottom‐up inventories.

#### 3.1.2 Burned Area

The two methods for calculating burned area using a bottom‐up approach operate under specific assumptions that could cause either an over or under estimation of carbon emissions. The active fire based method has the potential to overestimate burned area because it assumes all the area within the resolution of a single active fire detection is burned. Conversely, the burned area based method using MODIS burned area data products (MCD64A1) has been shown to underestimate burned area because of high omission error in grid cells with smaller proportions of burned area (Boschetti et al., 2019). A recent validation study that compares 500‐m resolution MODIS burned area products (MCD64A1) against 30‐meter resolution Landsat data found MODIS underestimated global burned area by 54% (Boschetti et al., 2019). However, MODIS burned area products also have a nontrivial level of uncertainty, ∼44% (Giglio et al., 2018).

Fuel2Fire, calculates burned area using the active fire approach, while GFED uses MODIS burned area data products. GFED4.1s attempts to address the known small fire driven burned area underestimation from MODIS using a supplementary algorithm known as the small fire boost (Randerson et al., 2012; Van Der Werf et al., 2017). We compare the GFED and Fuel2Fire burned area estimates in Figure S2, which are in good agreement for the western fires sampled during FIREX‐AQ (slope = 0.97, *r* = 0.93). This indicates that the differences in the two approaches to calculate burned area are not responsible for the low bias we see in GFED emission rate estimates.

#### 3.1.3 Combustion Completeness

All state‐of‐the‐art approaches to calculate combustion completeness rely on daily or monthly average observations, and therefore they cannot accurately estimate the pronounced subdaily changes in combustion completeness that occur throughout the diurnal cycle of fire activity. Instead, these methods assume combustion completeness can be estimated using observations averaged over large areas. Combustion completeness in the Fuel2Fire inventory is based on daily fire weather danger ratings, where higher levels of fire danger equate to higher consumption rates, while GFED models combustion completeness as a function of fuel type and fuel moisture conditions within the framework of a satellite‐driven biogeochemical model with a monthly temporal resolution.

In GFED, the average combustion completeness for temperate North American fires from 1997 to 2016 is 0.39 for standing fuel (all litter and biomass), while the average Fuel2Fire combustion completeness across all of the Western US wildland fires sampled during FIREX‐AQ is 0.5 for standing fuel. GFED underestimates *E*_*C*_ for almost every fire included in this analysis (Figure 3). This suggests the differences in the approaches to calculate combustion completeness could potentially account for ∼22% of the systematic low bias found in GFED *E*_*C*_ estimates. It has been shown that GFED average fuel consumption for temperate forest fires is 33% below measured values (Van Der Werf et al., 2017), however the low bias compared to observations was attributed to an anomalously high field based measurement of fuels from a temperate forest fire in Tasmania. Alternatively, it is possible that the low bias in GFED combustion completeness for temperate North America is a result of climate change causing a shift toward more favorable fire weather conditions that support enhanced fuel consumption (Abatzoglou & Williams, 2016), however additional field measurements of fuel consumption from Western US wildland fires from more recent years are needed to confirm this hypothesis.

#### 3.1.4 Fuel Loading

The complexity and variability of fuel type (or land cover) and loading are difficult to accurately represent and validate. High spatial resolution fuel databases, such as the FCCS database used in the Fuel2Fire inventory, are derived from a compilation of previous remote sensing studies, government databases, photos, in situ measurements, and expert opinion (Ottmar et al., 2007). The spatial resolution of FCCS is 30 m, but this resolution is achieved through the extrapolation of field‐based measurements to ecosystem scales. The extrapolation relies on a number of strong assumptions that infer the distribution and composition of fuels from the same, similarly aged, spectrally similar ecosystems are roughly spatially constant. However, fuels are constantly changing in response to seasonal, environmental, and anthropogenic forcing, but the laborious effort required to develop fuel databases severely restricts the rate at which they can be updated. As a result, fuel bed databases can remain unchanged and out of date for a number of years before updates are implemented. This delay can exacerbate the uncertainty and error in fuel loading estimates. A previous study compared FCCS with an extensive data set of USFS Forest Inventory and Analysis plot data (>10,000 plots) and discovered FCCS suffered from poor classification accuracy (Keane et al., 2013), which may explain the significant spread we see between emission rate estimates from Fuel2Fire versus the in situ measurement based approach. Model based estimates of fuel loading that rely on remote sensing observations of surface characteristics, like those used in GFED, are similarly challenged by the limited number of field measurements available to validate estimates. Potential bias stemming from fuel loading estimates can be negative or positive, depending on the accuracy of initial estimates and if the database or model correctly implements changes in fuel loading following ecosystem disturbance mechanisms including fire.

We discover an exceptional uncertainty in GFED fuel loading (111%), however the uncertainty in the FCCS fuels database used by Fuel2Fire is estimated to be much lower (∼70%) (Keane et al., 2013, Table S1). The likely cause for the persistent underestimation of GFED *E*_*C*_ estimates from Western US wildland fires stems from the differences in fuel loading estimates from the model used in GFED combined with a low bias in combustion completeness. The agreement we see in *E*_*C*_ estimates from Fuel2Fire versus the in situ based approach provides confidence in the use of high‐resolution fuels databases such as FCCS (Figure 2).

Previous studies aimed at quantifying uncertainty in the parameters used by bottom‐up inventories to calculate emissions have also identified fuel loading as a major source of uncertainty (French et al., 2004; Kennedy et al., 2020; Larkin et al., 2012; Prichard et al., 2019; Urbanski et al., 2011). Furthermore, fuel loading uncertainty likely fluctuates considerably as a function of vegetation type, due to scarce field validation studies for certain ecosystems and/or mapping errors. A recent study by Prichard et al. (2019) recommends using fitted distributions of fuel loading based on available data as an effort to capture the variability that exists in this parameter. The North American Wildland Fuels Database is an example of a geospatial database that provides these distributions along with robust uncertainty estimates (https://fuels.mtri.org/). Our results highlight the need for additional field validation studies to constrain fuel loading estimates and support the use of a fuel loading distribution as opposed to a single value.

### 3.2 Total PM Emission Rates

**Figure 4:**
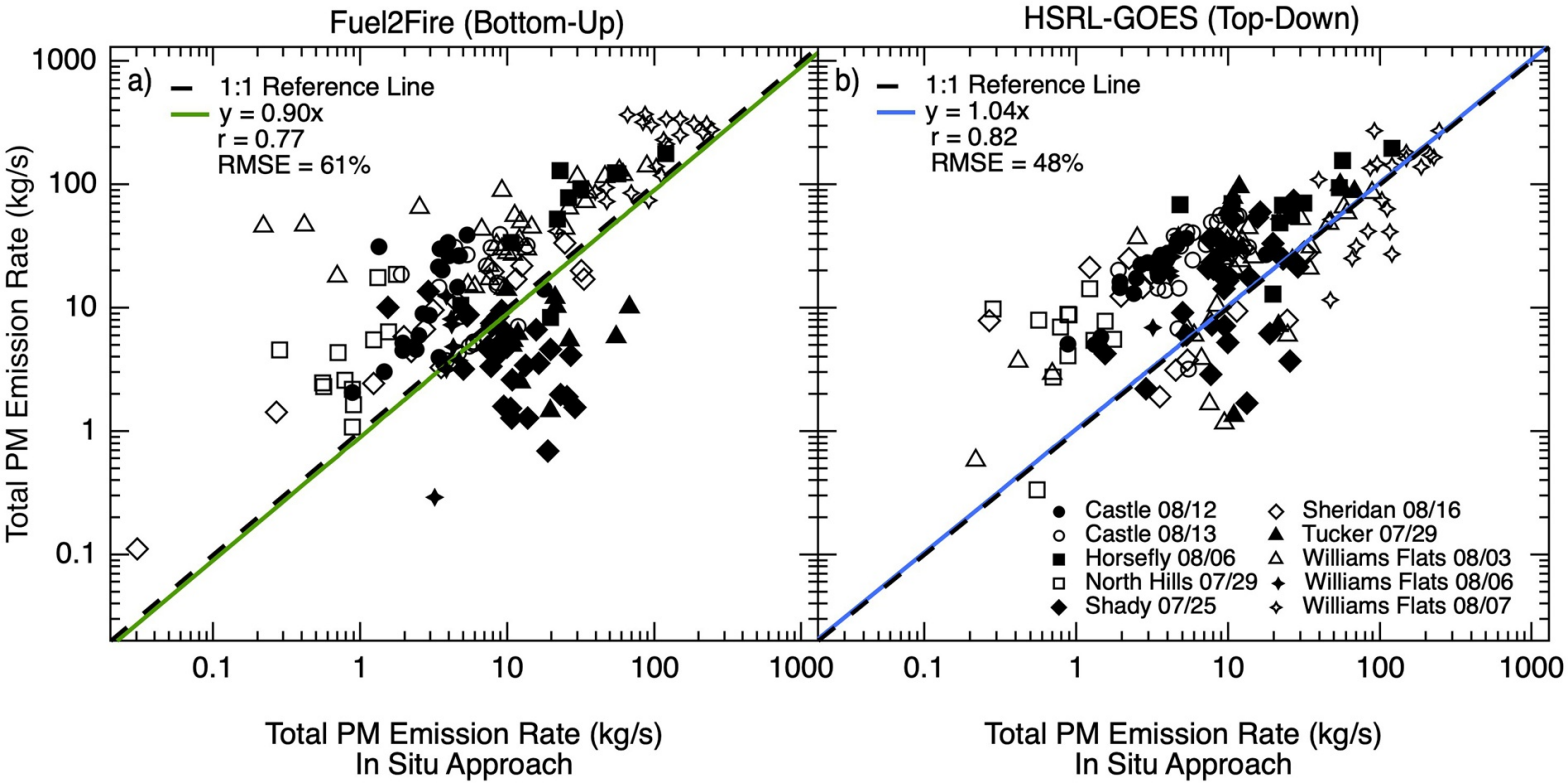
Relationship between total PM emission rates (*E*_PM_) derived from the high‐resolution bottom‐up approach (Fuel2Fire) versus in situ shown in panel a, and the same relationship between the high‐resolution top‐down aircraft approach (HSRL‐GOES) and the in situ approach shown in panel (b). Different markers correspond to specific sampling days for each fire and repeated markers correspond to different transects of the same fire for the given sampling day. The green line in panel a shows the reduced major axis regression with a forced zero intercept for Fuel2Fire *E*_PM_ estimates versus in situ, and the blue line in panel b shows the fit for the HSRL‐GOES *E*_PM_ estimates versus in situ. Legend gives the slope for the linear fit, Pearson's correlation coefficient (*r*), and root mean square error (RMSE).

We find the strong relationship between Fuel2Fire and the in situ based method persists for *E*_PM_ at a subplume scale, albeit with a similarly high level of scatter as shown in Figure 4a and Table 2 (slope = 0.90, *r* = 0.77, RMSE = 61%). We derive EF_PM_ from the in situ FIREX‐AQ measurements on a per transect basis in order to minimize the potential influence of emission factor uncertainty in Fuel2Fire *E*_PM_ estimates. The high level of spread in the data is likely an artifact of the uncertainty in *E*_*C*_ from Fuel2Fire caused by the biases and sources of uncertainty discussed in Section 3.1, most notably the impacts of fuel loading uncertainties. Additionally, this comparison is based on the assumption that the transport of fire emissions from the ground to the in situ transect is accurately modeled in both space and time.

**Table 2:** Reduced Major Axis Regression Slope (*m*), Pearson's Correlation Coefficient (*r*), and Root Mean Square Error (RMSE) for Particulate Mass (PM) Emission Rates (*E*_PM_) From Fuel2Fire and HSRL‐GOES Versus the In Situ Based Approach Per Fire

Fire name	Date flown	Fuel2Fire *E*_PM_			HSRL‐GOES *E*_PM_		
		*m*	*r*	RMSE	*m*	*r*	RMSE
Shady	07/25	0.13	0.44	13%	1.69	0.53	67%
North Hills	07/29	1.69	0.45	58%	6.27	0.55	30%
Tucker	07/29	0.10	0.61	39%	1.59	0.66	107%
Williams Flats	08/03	1.13	0.89	15%	1.07	0.84	149%
Williams Flats	08/06	0.59	0.07	116%	5.76	0.33	37%
Horsefly	08/06	1.70	0.63	627%	1.92	0.89	15%
Williams Flats	08/07	0.88	0.63	45%	0.94	0.69	87%
Castle	08/12	1.10	0.56	29%	3.54	0.73	18%
Castle	08/13	1.15	0.53	232%	4.48	0.71	232%
Sheridan	08/16	0.41	0.77	1529%	0.93	0.69	276%

*Note*. Fire name is given in the far left panel, followed by date flown

Figure 4b and Table 2 shows a significant relationship between *E*_PM_ calculated using HSRL‐GOES and the in situ approach at a sub‐plume scale (slope = 1.04, *r* = 0.82, RMSE = 48%). While there is a marginally lower level of scatter in this relationship as shown by the RMSE, HSRL‐GOES slightly overestimates *E*_PM_ on the lower end of the scale compared to the in situ approach. This overestimate implies from Equation 3 that either or both the GOES FRP and *C*_*e*_ for these fire transects are biased high, where it follows from Equation 8 that the latter may be influenced by a low estimate of the smoke MEE or a high estimate of the optical thickness. A high optical thickness bias might be due to the extrapolation of HSRL extinction to the surface for cases when the laser light fully attenuates; although, we note the bias is most significant for the lower emission rates, which might discount this hypothesis.

**Figure 5:**
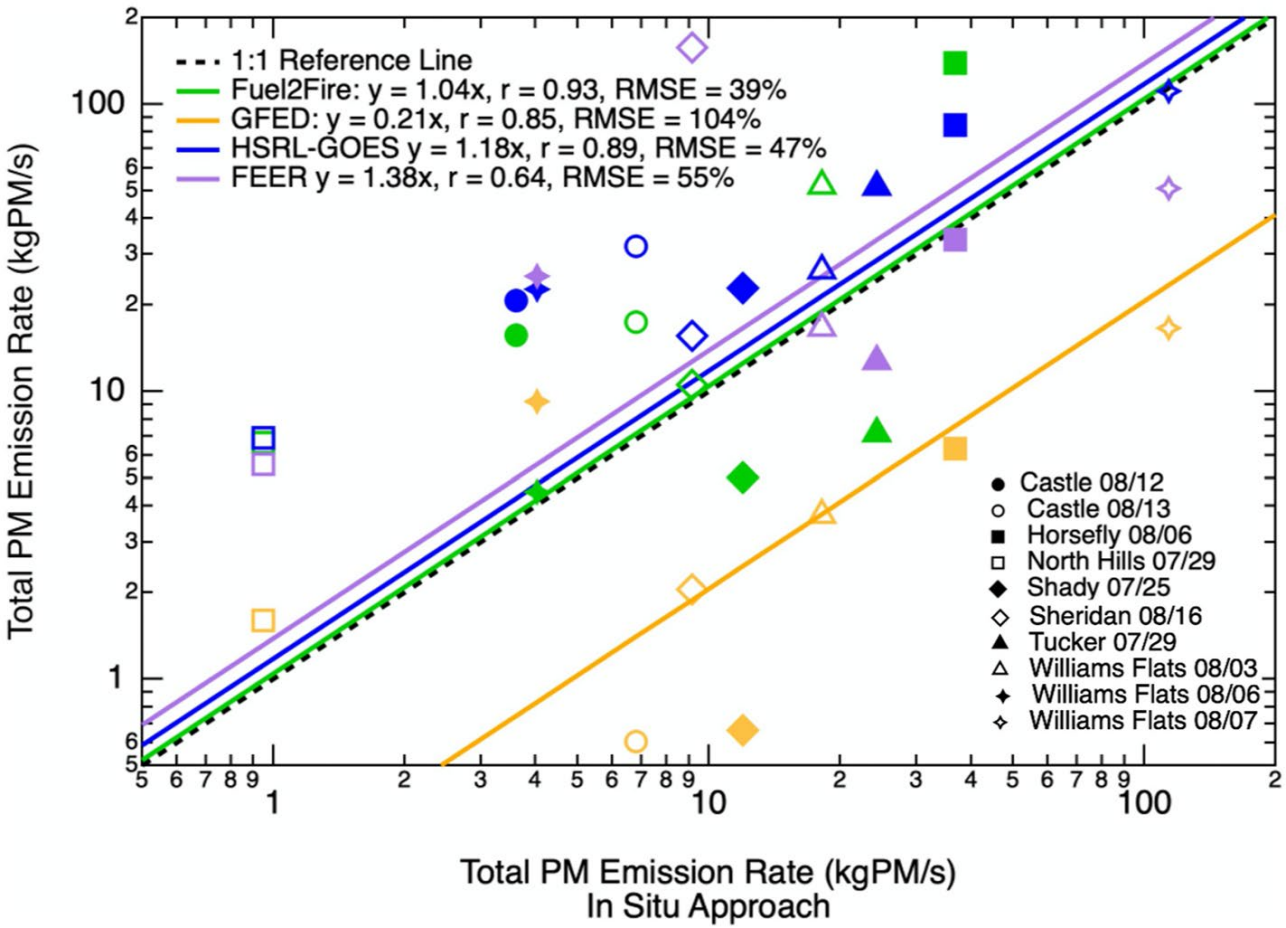
Daily fire average particulate mass (PM) emission rates (*E*_PM_) from Fuel2Fire, HSRL‐GOES, GFED, and FEER compared to estimates from the in situ approach. Different markers correspond to specific fires on specific sampling days. Green markers represent estimates from Fuel2Fire and the green line represents the reduced major axis regression with a forced zero intercept between Fuel2Fire estimates and in situ estimates. Blue markers and line represent HSRL‐GOES estimates and regression. Purple markers and line represent FEER estimates and regression. Orange markers and line represent GFED estimates and regression. The slope for the linear fit, Pearson's correlation coefficient (r), and root mean square error (RMSE) are given in the legend.

We find strong correlations between daily average *E*_PM_ estimates from the in situ approach versus estimates from both of the high‐resolution approaches, Fuel2Fire (slope = 1.04, *r* = 0.93, RMSE = 39%) and HSRL‐GOES (slope = 1.18, *r* = 0.89, RMSE = 47%) (Figure 5). The correlation is weaker and the spread is larger between the in situ based estimates and estimates from the lower‐resolution global inventories, GFED and FEER. The systematic low bias seen in GFED daily average *E*_*C*_ estimates is also seen for daily average *E*_PM_ for all but the fires with the lowest emission rates (slope = 0.21, *r* = 0.85, RMSE = 104%). FEER slightly underestimates *E*_PM_ from larger fires that have higher PM emission rates and overestimates *E*_PM_ from smaller fires that have relatively lower PM emission rates (slope = 1.38, *r* = 0.64, RMSE = 55%). FEER provides no *E*_PM_ estimates for the Castle fire on both days of sampling, and we exclude these zero estimates from this fire when computing the linear regression and correlation coefficient given their disproportionate weight in skewing the regression.

Global fire emissions inventories are known to significantly differ on *E*_PM_ estimates from temperate fires, especially in North America (Lu et al., 2019; Nikonovas et al., 2017; Pan et al., 2020). In the following sections, we use the high‐resolution airborne in situ measurements of smoke plumes collected during FIREX‐AQ to isolate the assumptions and variables responsible for the discrepancy and quantify their relative contributions.

#### 3.2.1 Emission Factors

Emission factors are used in bottom‐up approaches to convert carbon emissions to emissions of a specific trace gas or particulate species, and emission factor estimates usually come from compilation studies that include in situ measurements from wildland fires, prescribed fires and laboratory experiments (Akagi et al., 2011; Andreae, 2019; Andreae & Merlet, 2001; May et al., 2014). The use of such emission factors relies on the assumption that the most representative value can be approximated as the mean of all previous studies. In reality, emission factors are dynamic and vary as a function of combustion efficiency, which can spatiotemporally fluctuate for a given fire. Laboratory studies struggle to represent the complexity of a wildland fire and can disagree with in situ measurements, while in situ measurements are subject to sampling bias (Hodshire et al., 2019; Yokelson et al., 2013). For example, airborne based measurements tend to be limited to daytime sampling of well‐developed plumes that have risen to an altitude that is accessible by the aircraft. Consequently, these measurements may be biased toward flaming combustion because nighttime and/or smoldering emissions resulting from less energetic fire activity are likely not being sampled (Burling et al., 2011; Prichard et al., 2020; Wiggins et al., 2021). The suggested EF_PM_ for temperate forests from GFED is 17.6 g kg^−1^, and the mean EF_PM_ we calculated using FIREX‐AQ in situ airborne measurements is 15.8 ± 4.3 g kg^−1^, which is well within range of the suggested EF_PM_ from GFED. Our results suggest EF_PM_ does not strongly contribute to bias in bottom‐up emission rate estimates from Fuel2Fire or GFED for the fires sampled during FIREX‐AQ. However, we acknowledge this analysis focused exclusively on fires with well‐developed plumes that were sampled during the daytime, and thus may not be subject to EF_PM_ discrepancies that can occur, as a result of under sampled smoldering combustion.

#### 3.2.2 Smoke Emission Coefficient (*C*_*e*_)

**Figure 6:**
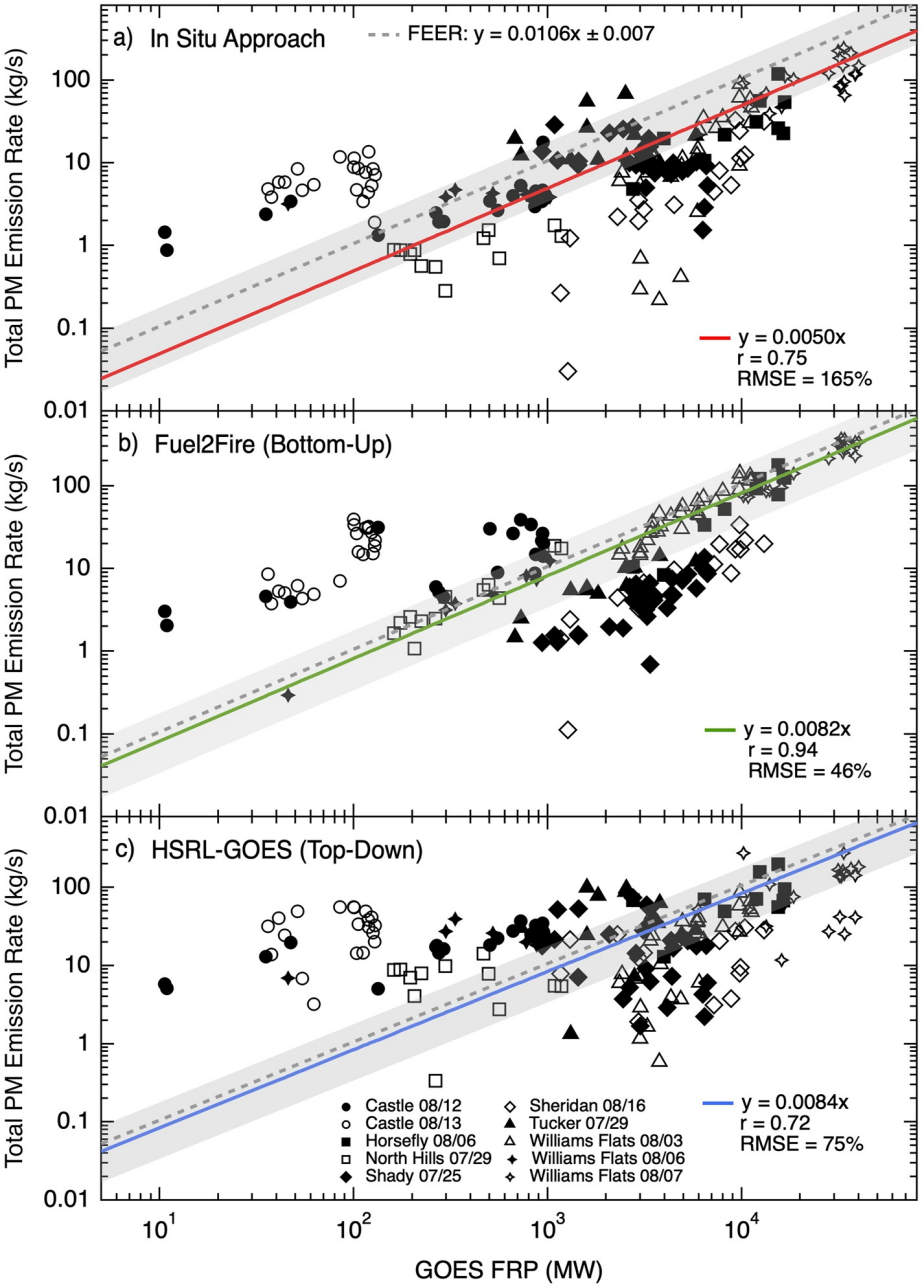
Relationship between GOES FRP and total particulate mass (PM) emission rates (*E*_PM_) derived from the in situ approach (panel a) and the same relationship for Fuel2Fire (panel b) and HSRL‐GOES (panel c). Different markers correspond to specific sampling days for each fire and repeated markers correspond to different transects of the same fire for the given sampling day. The red line shows the fit to a reduced major axis regression with a forced zero intercept for the GOES fire radiative power (FRP) versus in situ comparison, the green line shows the fit for Fuel2Fire, and the blue line shows the fit for HSRL‐GOES. The slope of each regression is equal to the smoke emission coefficient (*C*_*e*_). The dashed gray line is the *C*_*e*_ derived from FEER and the gray shading represents the corresponding uncertainty range. Legend gives the slope for the linear fit, correlation coefficient (r), and root mean square error (RMSE %).

Smoke emission coefficients used by top‐down inventories to convert FRP to *E*_PM_ are typically derived using multiple years of AOD and FRP observations, but here we use high‐resolution measurements from FIREX‐AQ to calculate *C*_*e*_ over a limited duration for a small number of fires. We find strong to moderate linear relationships between GOES FRP observations and the calculated emission rates from the high‐resolution in situ approach (*C*_*e*_ = 5.0 gPM MW^−1^, *r* = 0.75, RMSE = 165%), Fuel2Fire (*C*_*e*_ = 8.2 gPM MW^−1^, *r* = 0.94, RMSE = 46%), and HSRL‐GOES (*C*_*e*_ = 8.4 gPM MW^−1^, *r* = 0.72, RMSE = 75%) (Figure 6). Individual fires have significantly different *C*_*e*_, and vary depending on which approach was used to calculate *E*_PM_ (Table 3), which highlights the sensitivity and natural variability of this parameter. All three of the high‐resolution approaches estimate a lower *C*_*e*_ for the set of Western US wildland fires included in this study compared to the estimated *C*_*e*_ from FEER (10.6 gPM MW^−1^). However, the calculated *C*_*e*_ are within the large uncertainty (50%) of the *C*_*e*_ for western US fires derived from FEER, and the RMSE is substantial for the in situ approach and HSRL‐GOES.

**Table 3:** Reduced Major Axis Regression Slope (*m*), Pearson's Correlation Coefficient (*r*), and Root Mean Square Error (RMSE) for GOES Fire Radiative Power (FRP) Versus Total PM Emission Rates (*E*_PM_) for the In Situ Approach, Fuel2Fire, and HSRL‐GOES Per Individual Fire

Fire name	Date flown	In situ			Fuel2Fire			HSRL‐GOES		
		*M*	*r*	RMSE	*m*	*r*	RMSE	*m*	*r*	RMSE
Shady	07/25	0.0022	0.53	85%	0.001	0.80	148%	0.012	0.44	78%
North Hills	07/29	0.0011	0.77	57%	0.014	0.95	46%	0.015	0.61	40%
Tucker	07/29	0.0076	0.49	74%	0.003	0.89	55%	0.033	0.60	63%
Williams Flats	08/03	0.0039	0.69	934%	0.010	0.91	71%	0.011	0.69	139%
Williams Flats	08/06	0.0039	0.70	6%	0.010	0.96	491%	0.057	0.63	69%
Horsefly	08/06	0.0012	0.61	292%	0.008	0.84	311%	0.012	0.69	118%
Williams Flats	08/07	0.0040	0.67	21%	0.009	0.90	1%	0.008	0.45	18%
Castle	08/12	0.0060	0.58	4531%	0.027	0.68	209%	0.060	0.86	111%
Castle	08/13	0.0646	0.61	57%	0.204	0.65	147%	0.555	0.55	81%
Sheridan	08/16	0.0017	0.68	3154%	0.002	0.84	1199%	0.005	0.66	274%

*Note*. The slope is equal to the smoke emission coefficient (*C*_*e*_)

Fuel2Fire temporally distributes emissions using the diurnal cycle of GOES FRP observations, which explains the exceptionally strong linearity and correlation between GOES FRP and *E*_PM_ estimates in Figure 6b. HSRL‐GOES *E*_PM_ estimates shown in Figure 6c continue to have a slight high bias on the lower end of the scale. We find a high bias in *E*_PM_ for the Castle fire in all three high‐resolution approaches compared to what would be expected based on the overall campaign level relationship between FRP and emission rates (Figure 6). The Castle fire had the lowest average excess PM concentrations per transect out of all the fires included in this analysis. The elevated emission rates from all three approaches indicate GOES likely missed some of the FRP, likely due to low temperature smoldering or cloud cover, which is consistent with the low fire severity measured in postburn satellite data.

FEER uses MODIS FRP observations to calculate *C*_*e*_, but we use GOES FRP. There could be a potential offset between FRP observations between MODIS and GOES as a result of differences in instrument resolution and saturation levels as well as overpass time effects (Figure S3, Li et al., 2019; Xu et al., 2021). The coarse spatial resolution of GOES (2 km) limits its capability to detect cool or small fires with low FRP, and could result in an underestimation of FRP by up to 50% globally (Freeborn et al., 2008), which would explain the difference in *C*_*e*_ estimated using the high‐resolution approaches versus FEER. We use GOES FRP because of the exceptionally high time resolution (5–15 min) over the continental US versus the twice daily temporal resolution of MODIS or VIIRS. This allows for a more direct comparison between in situ measurements and remote sensing observations.

Previous studies have suggested *E*_PM_ and thus *C*_*e*_ calculated using MODIS AOD may be systematically biased low because of a discrepancy between observed AOD from MODIS versus AERONET and MISR, and/or because of invalid assumptions including the assumption of a constant MEE or the assumption of linear variation in FRP between Terra and Aqua overpass times (Lu et al., 2019; Nikonovas et al., 2017; Pan et al., 2020). Conversely, we find agreement between *E*_PM_ calculated independently of MODIS AOD and *E*_PM_ estimated from FEER, which relies on MODIS AOD observations. The results suggest MODIS AOD observations can be used to represent atmospheric aerosol mass loading of particulates emitted by Western US wildland fires, however, the high RMSE in the linear fits used to derive smoke emission coefficients in this study and the lack of agreement with more recent studies indicates more research is needed to address this issue.

##### 3.2.2.1 Mass Extinction Efficiency (MEE)

The conversion of FRP to PM assumes that variability in particle extinction, and thus AOD, is driven by changes in aerosol mass concentration rather than aerosol intensive properties. Estimates of particle mass extinction efficiency (MEE) are essential to the conversion of AOD to total PM. However, aerosol extinction and other optical properties depend on particle size, morphology, and chemical composition (Seinfeld & Pandis, 2006). The characteristics of biomass burning aerosols are known to vary with fuel type and combustion efficiency (McClure et al., 2020; Reid, Koppmann, et al., 2005; Reid, Eck, et al., 2005). Furthermore, the physical and optical properties of smoke aerosols rapidly evolve following emission as a result of photochemical aging and aerosol microphysical processes (Akagi et al., 2012; Cappa et al., 2020; Garofalo et al., 2019; Hodshire et al., 2019; May et al., 2014; Shingler et al., 2016). Particle evolution via these processes is additionally influenced by external factors, such as the fire size, rate of dilution, and background aerosol concentrations (Hodshire et al., 2019). The assumption that variability in AOD is entirely due to changes in particle concentration oversimplifies the complex interactions of smoke particle microphysical processes and photochemical aging. Some top‐down inventories attempt to reconcile this discrepancy by calculating a separate *C_e_* for each individual ecosystem. However, this is likely not sufficient to fully address the variability in smoke aerosol extinction that often occurs even in smoke plumes from fires within a single ecosystem type.

**Figure 7:**
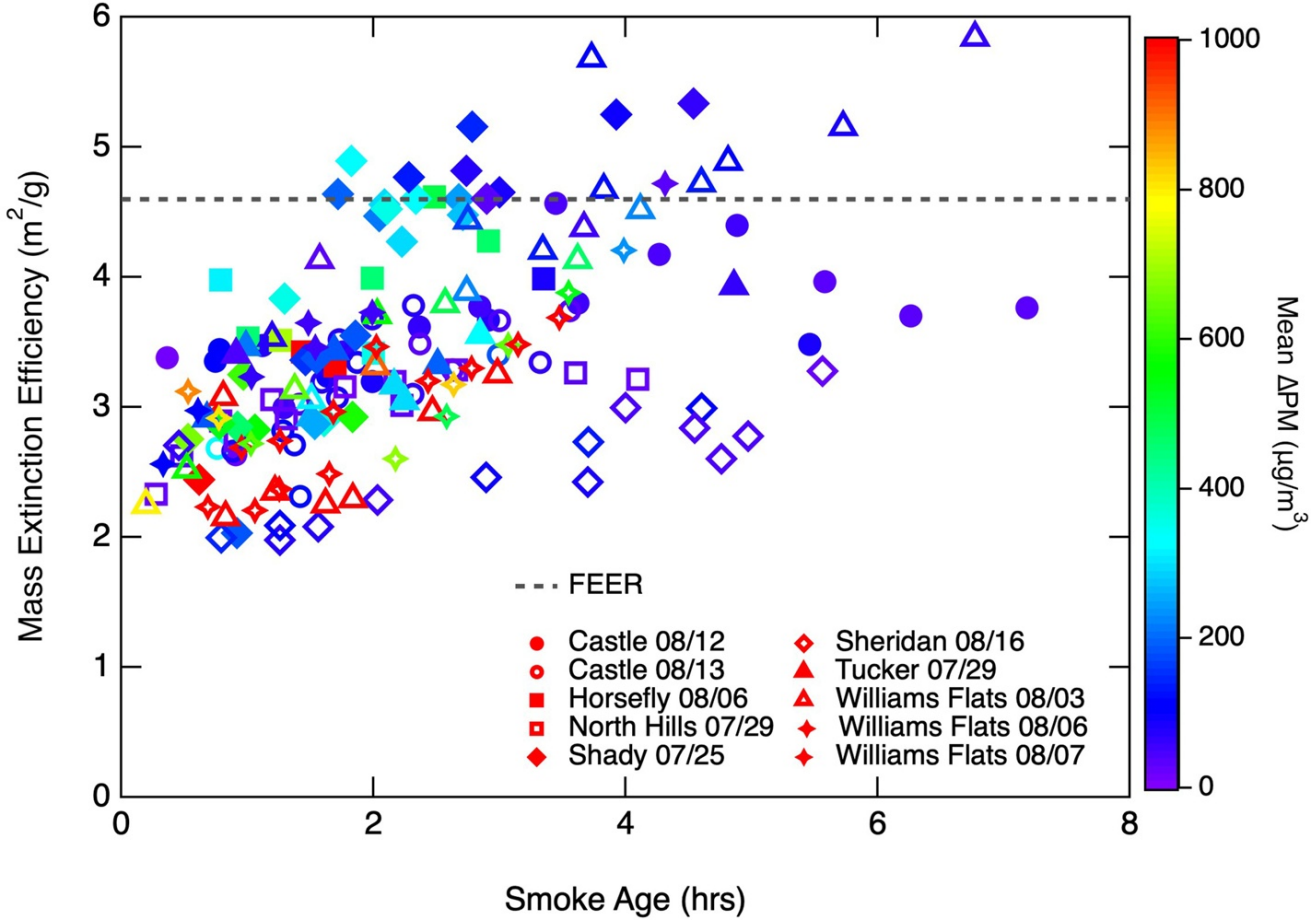
Mass extinction efficiency (MEE) versus smoke age per transect for each fire. Different markers correspond to specific sampling days for each fire and repeated markers correspond to different transects of the same fire for the given sampling day. Markers are colored as a function of transect mean excess particulate mass (PM) concentration. The constant MEE assumed by FEER is shown as the dashed black line for reference.

Top‐down inventories, including FEER, usually assume a constant MEE of 4.6 m^2^ g^−1^ based on a compilation of previous studies (Ichoku & Kaufman, 2005; Reid, Eck, et al., 2005). The compilation found MEE varied between 3.4 and 5.1 m^2^ g^−1^ for biomass burning particles of all ages across a diverse set of ecosystems (Figure S4) (Reid, Eck, et al., 2005). We find MEE values vary between 2 and 6 m^2^ g^−1^ for the FIREX‐AQ smoke plumes and that the MEE increases asymptotically as a function of smoke age (Figure 7). Our observations indicate MEE approaches the mean from previous studies as the smoke rapidly evolves in the early hours after emission. The rate at which MEE increases with smoke age is variable among the fires included in this analysis and does not appear to depend on the plume PM concentration. Our range of MEEs for smoke plumes from Western US wildland fires using high‐resolution in situ measurements is larger than what has been observed in previous studies. Our results emphasize the variability that can occur in smoke MEE, and suggest that the top‐down approach is likely more sensitive to MEE than previous studies imply. Additional measurements are needed to better understand the variability in MEE to ultimately improve parameterization of MEE with respect to smoke age. The use of a constant MEE could lead to a high bias for fires with lower excess PM concentrations and a low bias for fires with higher excess PM concentrations, which would explain the trend we see in Figure 5, where FEER underestimates fire *E*_PM_ from the most actively burning fires and overestimates *E*_PM_ from smaller, weaker fires.

##### 3.2.2.2 Instantaneous Observations of FRP and AOD

FEER uses daytime MODIS FRP and AOD observations to derive *C*_*e*_ and assumes that the FRP at the time of observation is directly related to the smoke plume AOD. However, fires have a clear, ecosystem dependent diurnal cycle with the time of peak fire activity depending on the specific landcover, geographic location, elevation, slope, aspect, and type of fire (e.g., wildland, prescribed, crown, and surface). FRP observations represent the instantaneous fuel consumption and corresponding emissions of a given fire, but AOD observations represent the total mass of aerosols emitted by a fire, including the time period when the fire was active before the satellite overpass time. The variability in FRP that occurs over the course of a day has a clear impact on the total mass of smoke particles in the plume as a function of downwind distance from the fire and wind speed, but polar orbiters, like MODIS, do not have the temporal resolution to quantify this relationship. As a result, *C*_*e*_ derived using FRP and AOD observed after the peak in diurnal fire activity are likely overestimated, while *C*_*e*_ derived using FRP and AOD observed prior to the peak may be slightly underestimated. The exact nature and magnitude of the potential bias would depend on a specific fire's diurnal cycle and the age of the smoke captured in the satellite observations of AOD. With respect to the calculation of *E*_PM_ using a predetermined *C*_*e*_, the time offset between MODIS overpass times and peak diurnal fire activity could similarly cause a bias. *E*_PM_ could potentially be biased high or low if the satellite overpass time occurred either before or after the peak in diurnal fire activity, and if the observed FRP was higher or lower than the daily average FRP.

A recent study by Mota and Wooster (2018) demonstrated fire emission rates can be calculated at a high temporal and spatial resolution (hourly and 0.05° × 0.05°, respectively) using a top‐down approach that relies on geostationary satellite observations of FRP from the Spinning Enhanced Visible and InfraRed Imager (SEVIRI) to avoid bias caused by inadequate sampling of a fire's diurnal cycle. We compare geostationary satellite observations of FRP from GOES that match the overpass times of MODIS with the average of all FRP observations over the course of a day for each fire to investigate potential bias in *E*_PM_ estimates from FEER. The Western US wildland fires sampled during FIREX‐AQ exhibited peak fire activity from 3 to 6 p.m. local time (Pacific/Mountain daylight time, UTC‐7/UTC‐6) (Wiggins et al., 2020). Meanwhile, local MODIS overpass times are ∼10:30 a.m. for the Terra satellite and ∼1:20 p.m. for Aqua. We find average GOES FRP at the time of the MODIS overpasses is double the daily average FRP from GOES (Figure S3), which could be partially responsible for the overestimation in FEER *E*_PM_ estimates for smaller fires that we see in Figure 5.

## 4 Summary and Conclusions

We present a comprehensive evaluation of total carbon and aerosol emission rate estimates computed using the methodologies and assumptions that are commonly employed by global inventories used by models. These emissions inventories have the monumental task of capturing the composition, magnitude, and temporal variability of fire emissions from nearly every ecosystem on Earth. They are critical for the representation of wildland fires in large‐scale models, and only recently have sufficiently comprehensive observational datasets become available to evaluate their performance. One such study is the 2019 joint NASA/NOAA FIREX‐AQ airborne mission. Here, we extend the methods and assumptions employed by emissions inventories to develop state‐of‐the‐art, high‐resolution emission rate estimates for each of the western FIREX‐AQ fires, which are based on detailed information garnered from ground, airborne, and satellite assets.

We discover excellent agreement between the high‐resolution emission rate estimates calculated using integrated airborne in situ and lidar observations and the high‐resolution top‐down (HSRL‐GOES) and bottom‐up (Fuel2Fire) estimates at unusually high sub‐plume spatiotemporal resolution. While there is considerable scatter in the one‐to‐one plots comparing Fuel2Fire to the airborne in situ data, the emissions rate estimates for both total carbon and PM are not consistently biased between these methodological approaches. HSRL‐GOES appears to slightly overestimate *E*_PM_ toward the lower end of the observed range of variability (which appears to also scale with FRP). Emission rate estimates calculated using the lower resolution global fire emissions inventories, FEER and GFED, have a weaker relationship with the high‐resolution approaches and show evidence of systematic bias, which is most apparent for GFED.

We discuss, in detail, the key assumptions employed by bottom‐up approaches and conclude that the strong performance of the Fuel2Fire inventory stems from detailed information about fuel type and loading that are parameterized with significant uncertainty in the global inventories. Combustion completeness is likely underestimated in GFED for fires in temperate North America, and estimates could potentially be improved by utilizing daily fire weather danger ratings instead of relying entirely on model estimates of fuel moisture. In addition, we note that the high‐temporal resolution of the Fuel2Fire data set also allows it to capture the entire diurnal cycle of the fire activity, which also serves to improve its predictive skill. This hints that the high temporal resolution of geostationary satellite observations of FRP could be used to correct the bias caused by satellite overpass times. With respect to top‐down approaches, we find a larger range in MEE for this small subset of Western US fires than what has been reported in a compilation of previous studies that includes MEE from fires in a diverse selection of global ecosystems. The high‐resolution top‐down approach (HSRL‐GOES) allowed for the application of variable MEE obtained from sub‐plume in situ measurements. HSRL extinction measurements of the smoke plumes sampled during FIREX‐AQ combined with geostationary satellite observations of FRP offered an exceptionally detailed measure of AOD and FRP associated with the smoke plume. The use of a constant MEE to convert AOD to PM in top‐down approaches combined with bias from assumptions related to instantaneous observations of FRP and AOD are likely responsible for the underestimation in FEER *E*_PM_ for larger fires and overestimation for smaller fires.

Finally, it is important to note that it is not yet computationally practical or feasible for global fire emissions inventories to achieve the level of complexity and detail in the high‐resolution approaches presented here. We use these approaches to investigate discrepancies between top‐down and bottom‐up *E*_PM_ estimates for Western US wildland fires. However, this collection of fires represents only a small subset of the total number of fires that burn every year in the Western US and may not be a perfect representation of the complexity that can exist in fire emissions. In short, we have the luxury of evaluating the skill of the global emissions inventories for a small subset of wildland fires for which we have unprecedentedly comprehensive data, but we would be wise to remember that the goal of global emissions inventories is to represent all fires reasonably well rather than to represent a few fires perfectly. Consequently, it may be premature to adopt new values for, for example, the smoke emission coefficient based solely on the FIREX‐AQ data set. Our analysis does emphasize areas of large uncertainty that may be improved. One is the estimate of fuel type and loading that likely contributes to the scatter we see in the bottom‐up emission rate estimates from GFED and Fuel2Fire. Burned area and aerosol mass emission factors do not appear to be large sources of uncertainty as there is good agreement seen for both GFED and Fuel2Fire for both of these metrics. The importance of the high‐temporal resolution observations of both FRP and smoke AOD afforded by the geostationary satellites currently in orbit cannot be overstated, as a lack of complete orbital coverage is also likely to be a strong contributor to the inventory emissions underestimates. The use of a constant MEE to convert AOD to PM should be revisited in light of the much higher variability we find in MEE observations for such a limited number of fires, which accentuates the need for additional measurements of this key variable. In summary, both top‐down and bottom‐up global fire emissions inventories suffer from assumptions that may hold true in the aggregate, but break down on an individual fire basis. The strong agreement that we show here between the high‐resolution approaches holds promise for future fire emissions inventories as advances in remote sensing, improved computational efficiency, and a more complete understanding of fire behavior begin to offer opportunities to increase the accuracy and resolution of global fire inventories.

## Conflict of Interest

The authors declare no conflicts of interest relevant to this study.

## Supporting information

Supporting Information S1

## Data Availability

All FIREX‐AQ data are publicly available online at: https://www-air.larc.nasa.gov/cgi-bin/ArcView/firexaq.

## Appendix A List of Variables and Common Units

### A.1

**Table:**

$\alpha_{t}$	HSRL extinction coefficient	km^−1^
*Α*_P,FEER_	FEER pixel area	km^2^
*Α*_P,GFED_	GFED pixel area	km^2^
AOD	Aerosol optical depth	unitless
*β*	HSRL backscatter ratio	${\text{km}}^{-1}\;{\text{sr}}^{-1}$
BA	Burned area	m^2^
CC	Combustion completeness	%
*C*_*e*_	Smoke emission coefficient	gPM MW^−1^
Δ*C*	Excess mass concentration of C	μgC m^−3^
ΔPM	Excess mass concentration of PM	μgPM m^−3^
Δ*t*	HSRL curtain pixel width	s
Δ*z*	HSRL curtain pixel height	m
*E*_*C*_	Emission rate of total carbon	kgC s^−1^
${\hat{E}}_{C}$	Area‐normalized emission rate of total carbon	kgC m^−2^ s^−1^
*E*_PM_	Emission rate of total PM	kgPM s^−1^
${\hat{E}}_{\text{PM}}$	Area‐normalized emission rate of total PM	kgPM m^−2^ s^−1^
EF_PM_	Particle mass emissions factor	gPM kg biomass consumed^−1^
*F*_*C*_	Mass fraction of carbon in the fuel	gC kg biomass consumed^−1^
FL	Fuel loading	g biomass m^−2^
FRP	Fire radiative power	MW
*H*	Plume vertical thickness	m
MEE	Particle mass extinction efficiency	m^2^ g^−1^
$\bar{MEE}$	Aircraft transect‐average MEE	m^2^ g^−1^
PM	Particle mass concentration	μgPM m^−3^
$\bar{GS}$	Transect‐average DC‐8 aircraft ground speed	m s^−1^
$\bar{WS}$	Aircraft transect‐average wind speed	m s^−1^

*Note*. Units are included here only as examples and do not consider any unit conversions that may be necessary for the equations given in the text.
