# Supplementary material for: Reconciling Assumptions in Bottom‐Up and Top‐Down Approaches for Estimating Aerosol Emission Rates From Wildland Fires Using Observations From FIREX‐AQ
Source: J Geophys Res Atmos. 2021 Dec 10;126(24):e2021JD035692. doi: 10.1029/2021JD035692 (PMC9286562; doi:10.1029/2021JD035692)
Supplement: Supplementary file 1 — Supporting Information S1 [file JGRD-126-0-s001.docx]

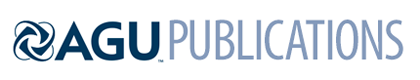


*Journal of Geophysical Research: Atmospheres*

Supporting Information for

**Reconciling Assumptions in Bottom-up and Top-down Approaches for Estimating Aerosol Emission Rates from Wildland Fires using Observations from FIREX-AQ**

**E. B. Wiggins^1,2^, B. E. Anderson^2^, M. D. Brown^2,3^, P. Campuzano-Jost^4^, G. Chen^2^, J. Crawford^2^, E. C. Crosbie^2,3^, J. Dibb^5^, J. P. DiGangi^2^, G. S. Diskin^2^, M. Fenn^2,3^, F. Gallo^1,2^, E. M. Gargulinski^6^, H. Guo^4^, J. W. Hair^2^, H. S. Halliday^7^, C. Ichoku^8^, J. L. Jimenez^4^, C. E. Jordan^2,6^, J. M. Katich^4,9^, J. B. Nowak^2^, A. E. Perring^10^, C. E. Robinson^2,3^, K. J. Sanchez^1,2^, M. Schueneman^4^, J. P. Schwarz^9^, T. J. Shingler^2^, M. A. Shook^2^, A. J. Soja^2,6^, C. E. Stockwell^4,9^, K. L. Thornhill^2,3^, K. R. Travis^2^, C. Warneke^9^, E. L. Winstead^2,3^, L. D. Ziemba^2^, and R. H. Moore^2^**

^1^NASA Postdoctoral Program, Universities Space Research Association, Columbia, MD

^2^NASA Langley Research Center, Hampton, VA

^3^Science Systems and Applications, Inc., Hampton, VA

^4^CIRES, University of Colorado Boulder, Boulder, CO, USA

^5^Earth Systems Research Center, University of New Hampshire, NH, USA

^6^National Institute of Aerospace, Hampton, VA

^7^Environmental Protection Agency, Research Triangle, NC, USA

^8^College of Arts and Sciences, Howard University, Washington, DC, USA

^9^NOAA Chemical Science Laboratory, Boulder, CO, USA

^10^Department of Chemistry, Colgate University, Hamilton, NY, USA

**Contents of this file**

Figures S1 to S4

Table S1


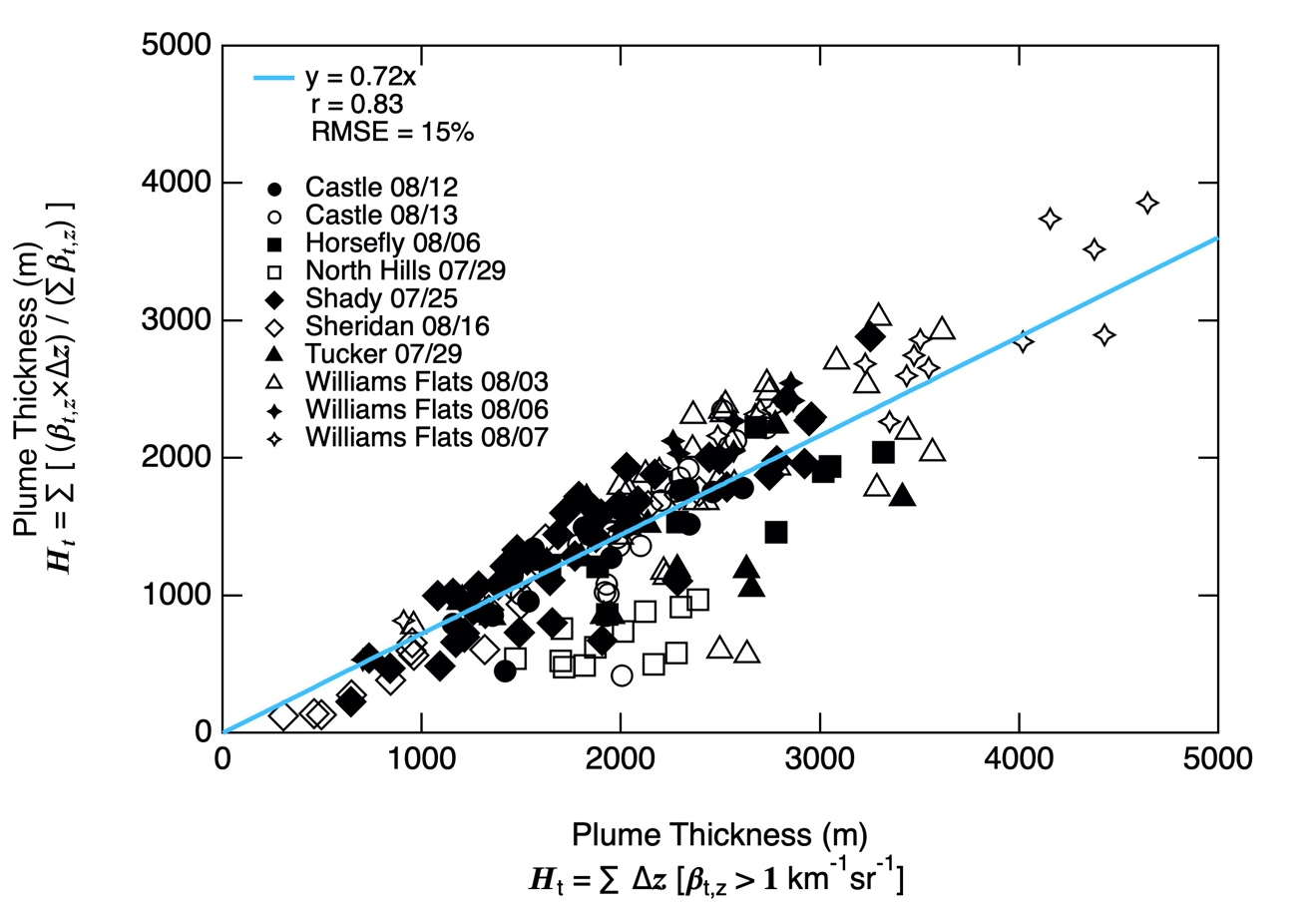


Figure S1. Relationship between two different methods to calculate plume thickness per transect for each fire. X-axis shows plume thickness calculated using equation 6 and y-axis shows plume thickness calculated as a function of the backscatter coefficient distribution throughout the HSRL curtain. Different markers correspond to specific sampling days for each fire and repeated markers correspond to different transects of the same fire for the given sampling day. Blue line shows the fit to a reduced major axis regression with a forced zero intercept. Pearson’s correlation coefficient (r) and root mean square error (RMSE) are given in the legend.


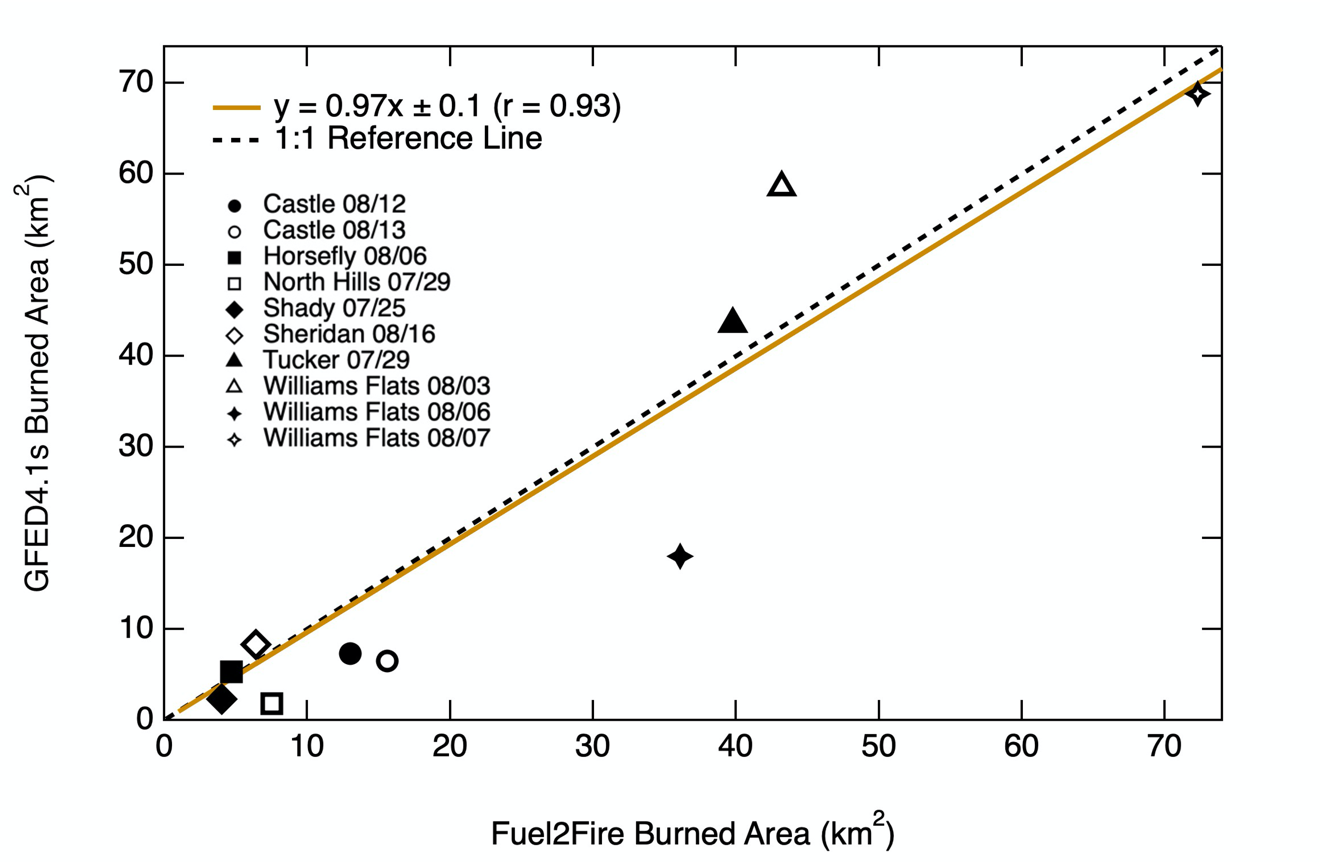


**Figure S2**. Total daily burned area per fire from Fuel2Fire versus GFED4.1s. Different markers correspond to specific fires on specific sampling days. The brown line shows the fit to a reduced major axis regression with a forced zero intercept. The slope and correlation coefficient are given in the legend. The black dashed line shows a perfect 1:1 relationship for reference.

**
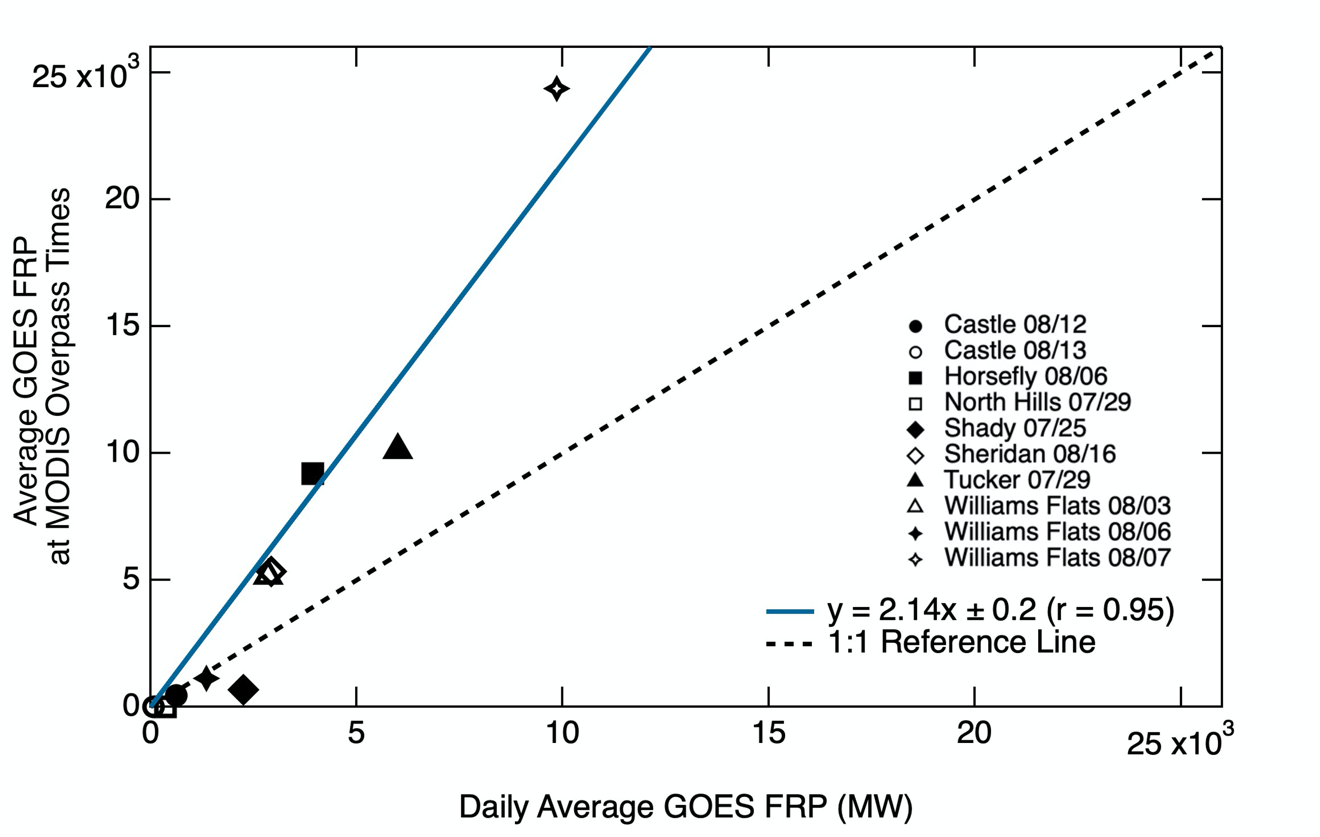
**

**Figure S3.** Daily average GOES FRP observations per fire versus average GOES FRP observations within 30 mins of the overpass times for MODIS onboard both Aqua and Terra per fire. Different markers correspond to specific fires on specific sampling days. The teal line shows reduced major axis regression line with the slope and correlation coefficient given in the legend. The black dashed line shows a perfect 1:1 relationship for reference.

**
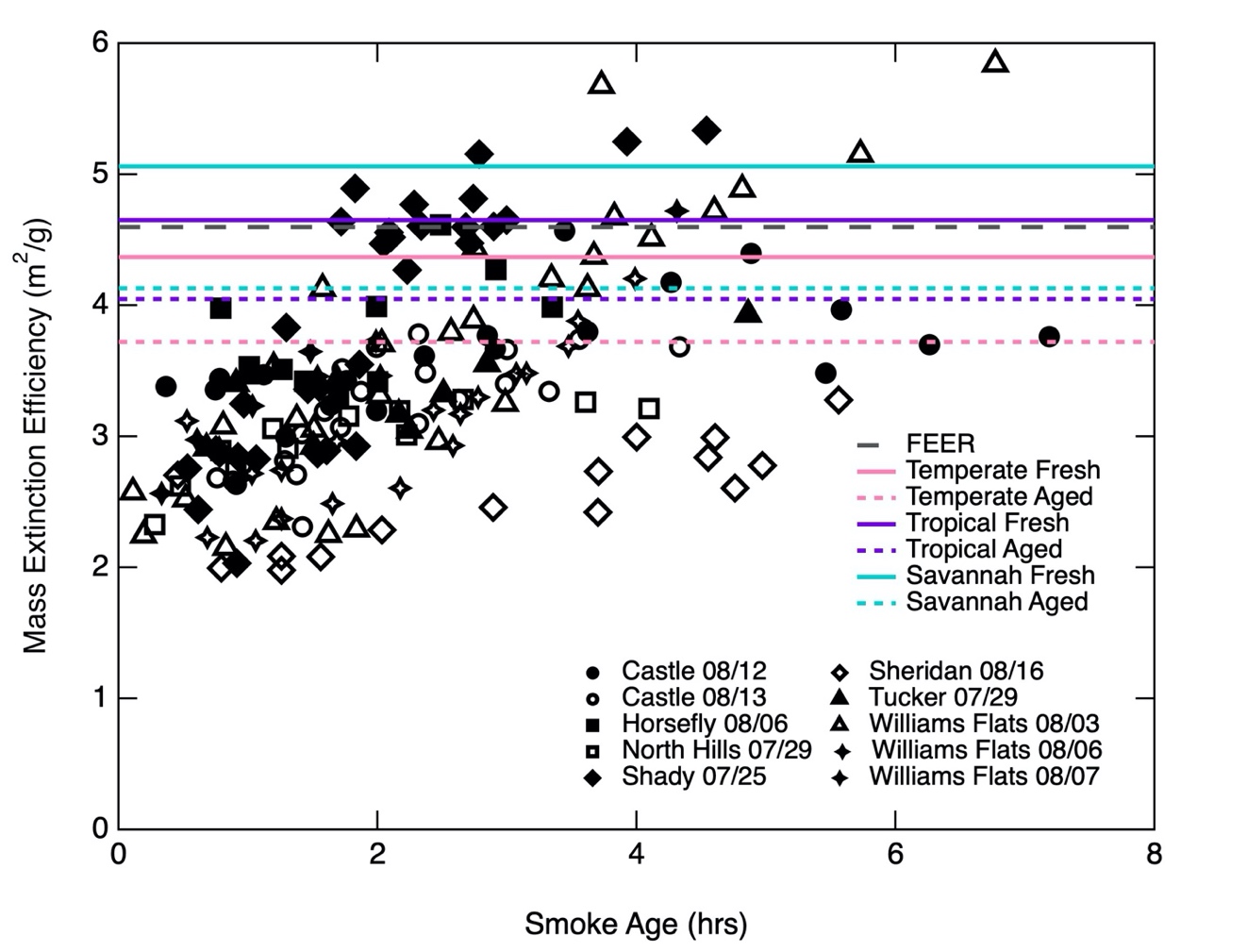
**

**Figure S4.** Mass extinction efficiency (MEE) calculated using in situ aircraft measurements for each fire per transect versus smoke age. Different markers correspond to specific sampling days for each fire and repeated markers correspond to different transects of the same fire for the given sampling day. Colored lines represent ecosystem average mass extinction efficiency for biomass burning particles taken from Reid et al. (2005b). Solid lines show MEE from fresh smoke (less than one day old) and dashed lines show MEE calculated using aged smoke (older than one day). The assumed MEE used by FEER is shown as the black dashed line.

| Approach | Variable Relative Error | | | μ ± σ | IQR | Reference |
| --- | --- | --- | --- | --- | --- | --- |
| GFED | δE_PM_ = 126% | δE_C_ = 120% | δBA = 44% | - | - | Giglio et al. (2018) |
|  |  |  | δFL = 111% | 75 ± 83 | 130 | Van Leeuwen et al. (2014) |
|  |  |  | δCC = 11% | 79 ± 9 | 14.0 | Van Leeuwen et al. (2014) |
|  |  |  | δFC = 10% | 500 ± 5 | - | Akagi et al. (2011) |
|  |  | δEF_PM_ = 36% | | 17.6 ± 6.4 | 7 | van der Werf et al. (2017) |
| FEER | δE_PM_ = 78% | δC_e_ = 73% | | 0.011 ± 0.008 | 0.008 | Ichoku and Ellison (2014) |
|  |  | δFRP = 27% | | - | - | Freeborn et al. (2014) |
| In Situ | δE_C_ = 66%  δE_PM_ = 75% | δWS = 17% | | 6 ± 1 | 4 | FIREX-AQ Observations |
|  |  | δGS = 3% | | 154 ± 5 | 16 |  |
|  |  | δHt = 28% | | 2121 ± 594 | 593 |  |
|  |  | δΔC = 56% | | 0.009 ± 0.005 | 0.009 |  |
|  |  | δΔPM = 67% | | 0.0006 ± 0.0004 | 0.001 |  |
| Fuel2Fire | δE_PM_ = 67% | δE_C_ = 55% | | 1322 ± 729 | 510 | Fuel2Fire (Internal) |
|  |  | δEF_PM_ = 38% | | 16 ± 6 | 7 | FIREX-AQ Observations |
| HSRL-GOES | δE_PM_ = 78% | δC_e_ = 67% | | 0.006 ± 0.004 | 0.005 | FIREX-AQ Observations |
|  |  | δFRP = 40% | | - | - | Li et al. (2020) |

Table S1. Relative uncertainty (δ) given as a percentage for E_PM_ and E_C_ (when available) derived using GFED, FEER, in situ measurements, Fuel2Fire, and HSRL-GOES. From left to right the dependent variables are broken down into the individual independent variables required for their calculation. Relative uncertainty for each independent variable is calculated as the standard deviation (σ) divided by the mean (μ), and relative uncertainty for each dependent variable is computed by error propagation through the equation by which they are defined. If the mean and standard deviation are not available, the relative uncertainty for a variable is taken directly from the corresponding reference. Mean, standard deviation, and interquartile range (IQR) are derived from aircraft observations during smoke plume transects and averaged over all the Western US wildland fires included in this study or calculated based on data from previous studies when available.
